# Small anion-assisted electrochemical potential splitting in a new series of bistriarylamine derivatives: organic mixed valency across a urea bridge and zwitterionization

**DOI:** 10.3762/bjoc.15.220

**Published:** 2019-09-24

**Authors:** Keishiro Tahara, Tetsufumi Nakakita, Alyona A Starikova, Takashi Ikeda, Masaaki Abe, Jun-ichi Kikuchi

**Affiliations:** 1 Department of Material Science, Graduate School of Material Science, University of Hyogo, 3-2-1, Kouto, Kamigori, Ako, Hyogo 678-1297, Japan; 2 Graduate School of Materials Science, Nara Institute of Science and Technology, 8916-5, Takayama, Ikoma, Nara 630-0192, Japan; 3 Institute of Physical and Organic Chemistry, Southern Federal University, pr. Stachki 194/2, Rostov on Don, 344090, Russian Federation

**Keywords:** anion binding, electrochemistry, hydrogen bonding, triarylamine, urea, zwitterionic mixed valency

## Abstract

We report the synthesis of a new bistriarylamine series having a urea bridge and investigate its mixed-valence (MV) states by electrochemical and spectroelectrochemical methods. We found that the supporting electrolytes had unusual effects on potential splitting during electrochemical behavior, in which a smaller counteranion thermodynamically stabilized a MV cation more substantially than did a bulky one. The effects contrary to those reported in conventional MV systems were explained by zwitterionization through hydrogen bonding between the urea bridge and the counteranions, increasing the electronic interactions between two triarylamino units. Furthermore, we clarified the intervalence charge transfer characteristics of the zwitterionic MV state.

## Introduction

Mixed-valence (MV) compounds have received increasing attention from the viewpoint of fundamental research on intramolecular electron transfer phenomena and application in molecular devices [1–5]. The radical cations of bistriarylamine derivatives bis(NAr_3_) are well-known MV compounds having π-conjugated bridges (where NAr_3_ = triarylamine) [6–18]. These studies focused on evaluating the intervalence charge transfer (IVCT) transition near-infrared (NIR) absorption from NAr_3_ to NAr_3_^•+^ units [1–5]. The IVCT absorptions of MV compounds are generally more pronounced in organic species [19–20] than in their inorganic counterparts. The strong IVCT characteristics of the bis(NAr_3_)^•+^ radical cations are well documented due to their good availability through common N–C bond-forming reactions and the stability of the NAr_3_^•+^ unit [6–18]. Photoswitchable mixed valency has recently been demonstrated with bis(NAr_3_)^•+^ radical cations having dithienylethene bridges [21–22]. This was achieved by a regulation of the π-conjugation length through photoinduced formation/dissociation of σ bonds in the bridge, which was accomplished with changeovers from a localized system (class I) to a moderately delocalized one (class II), as well as and from moderately delocalized one to a highly delocalized one (class III) [1–5]. However, attempts to change the MV characteristics by manipulating the bridge moieties through intermolecular interactions have not been reported for bis(NAr_3_) derivatives.

**Figure 1:**
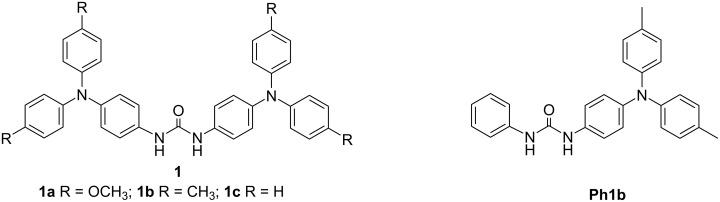
Structures of target compounds **1** and reference compound **Ph1b**.

Redox stimuli are a promising trigger to directly change the charge distributions of molecules and assemblies, potentially allowing tuning of the strength of non-covalent interactions, including hydrogen bonds (H-bonds) [23–26]. In this context, a number of redox-active compounds bearing H-bond donors and acceptors were investigated to realize electrochemically controlled H-bonding [23–36]. Especially, oxidation-active ureas are an important class of such compounds [25–26]. In the neutral state, ureas can provide two NH protons for multiple H-bonding, as often used for anion recognition [27–29]. In the oxidized state, the enhanced acidity of NH protons can increase the strength of H-bonds and give them more dynamic properties, which can be useful for refined designs of supramolecular systems [30] and proton-coupled electron-transfer systems [31–40]. In contrast to the vast majority of oxidation-active ureas, those having two redox centers at both ends have not received attention except for 1,3-bis(ferrocenyl)urea **FcFc** [41–42], a cyclometalated diruthenium complex [43], and bis(NAr_3_) counterparts, including **1a** (Figure 1) [44]. The electrochemically control of H-bonding would have a dramatic impact on the field of mixed valency, which is striving to utilize charge delocalization for molecular devices. However, there is a lack of basic knowledge about the influence of an excess of supporting electrolytes on the thermodynamic stability of urea-bridged MV species. In this study, we report the synthesis and characterization of a new series of urea-bridged bis(NAr_3_) derivatives **1** and investigate their MV states by electrochemical and spectroelectrochemical methods. We found that the supporting electrolytes have unusual effects on the thermodynamic stability of MV ions in terms of bulkiness of the counterion. Furthermore, we clarified the IVCT characteristics of the zwitterionic organic MV state.

## Results and Discussion

### Synthesis and characterization of **1** in the neutral form

The target compounds **1** with different substituents R at the *para*-position of the benzene adjacent to the nitrogen centers were synthesized to tune the oxidation potential of the nitrogen centers (Figure 1). According to a previously reported method for the synthesis of ureas [45], symmetric ureas **1** were synthesized from the corresponding amines with triphosgene. An unsymmetrical reference urea having a NAr_3_ moiety, **Ph1b**, was also synthesized. The new compound series was characterized by ^1^H NMR and EIMS (Figures S1–S4 in Supporting Information File 1). In the DFT-optimized structures of **1**, the ureylene moiety and the phenyl groups on both sides are almost coplanar with N···N distances between the NAr_3_ moieties of more than 13 Å (Figure S5 and Table S1 in Supporting Information File 1). Further investigations were not performed for **1c** because the solubility of this compound in aprotic solvents including CH_2_Cl_2_ were extremely low.

**Figure 2:**
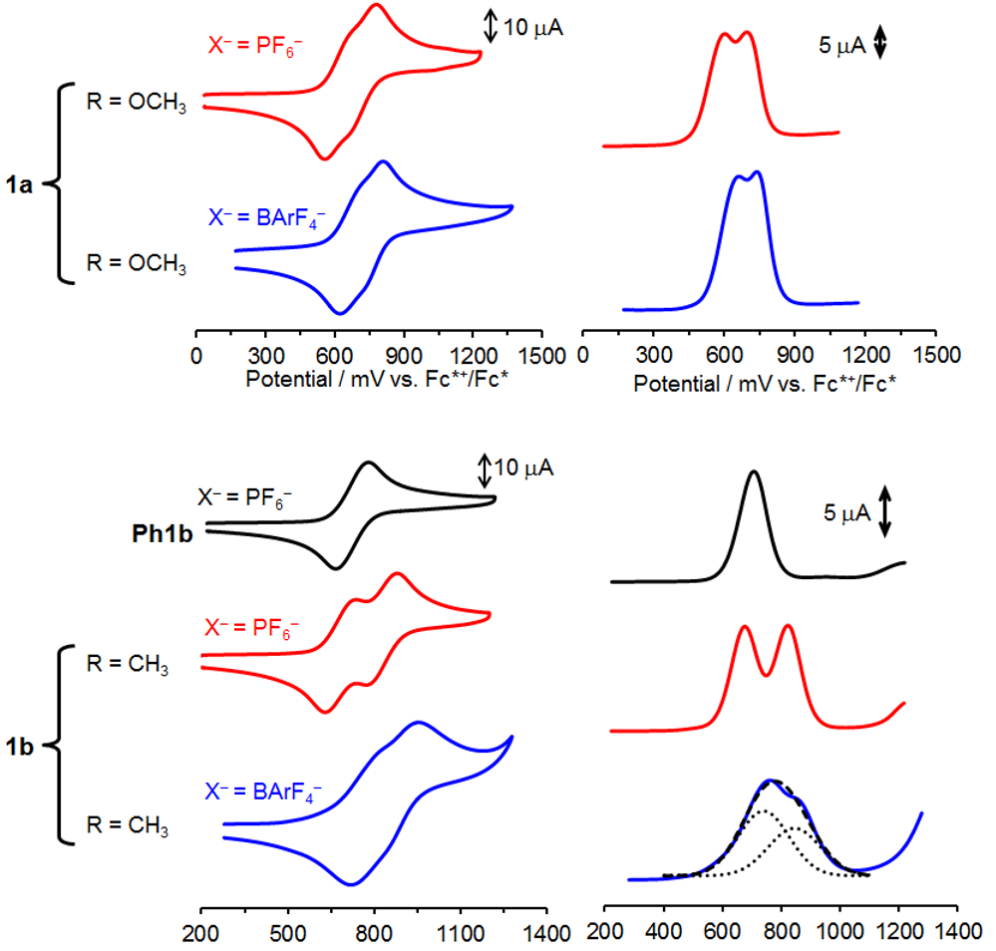
Cyclic voltammograms (left) and differential pulse voltammograms (right) of (top) **1a**, (middle) **Ph1b,** and (bottom) **1b** (1.0 mM) in CH_2_Cl_2_ containing *n*-Bu_4_NX (0.10 M). Scan rate: 100 mV s^−1^. For the differential pulse voltammogram of **1b** with BArF_4_^−^, Gaussian deconvolution (black dotted line) and the sum (black dashed line) were also shown.

The electrochemical behavior of **1a** and **1b** having two chemically equivalent NAr_3_ units was investigated by cyclic voltammetry and differential pulse voltammetry. Interestingly, two reversible waves were observed for **1b** in CH_2_Cl_2_ containing 0.10 M *n*-Bu_4_NPF_6_ (Figure 2, bottom, and Table 1 and Table S3 in Supporting Information File 1). In contrast, the reference urea **Ph1b** exhibited one reversible wave corresponding to the oxidation of the NAr_3_ unit, indicating that further oxidation of **Ph1b^+^** to the NAr_3_^2+^ species did not proceed in the potential range applied. The *E*_1/2_^1^ and *E*_1/2_^2^ values for **1b** are 28 mV lower and 116 mV higher, respectively, than the redox potential for **Ph1b**, affording a large split in the redox potentials of 144 mV. These results clearly indicate the consecutive oxidation of the NAr_3_ units in **1b** and the formation of the cationic and dicationic species, **1b^+^** and **1b^2+^** (Equation 1). It is notable that the organic MV species **1b^+^** has a relatively large comproportionation constant (*K*_c_), in the order of 10^2^ in the polar medium (CH_2_Cl_2_/*n*-Bu_4_NPF_6_), whereas small potential splitting values were often reported for general MV compounds [46–52]. The *E*_1/2_^1^ value for **1a** decreases by 90 mV compared with that for **1b**. This substituent effect on the oxidation potential is typical for the reported NAr_3_ system [53]. Because the electron-donating group of the NAr_3_ unit destabilizes the MV state, the potential splitting for **1a** is 50 mV smaller than that for **1b** (Figure 2, top). Similar substituent effects were previously reported for bis(NAr_3_) derivatives having π-conjugated bridges [8–9]. Judging from the long N···N distances (e.g., 13.16 Å for **1b**^+^) in the DFT-optimized structure (Figure S5 in Supporting Information File 1), the through-space electrostatic interactions between the NAr_3_ units could not contribute to the relatively large thermodynamic stability of **1a**^+^ and **1b**^+^. This indicated that the common stabilization mechanism for the covalently linked conventional MV species is applicable to the present urea-bridged MV species **1^+^**.

**Table 1:** Electrochemical data for **1** in CH_2_Cl_2_.^a^

Compounds	X^−^	*E*_1/2_^1^	*E*_1/2_^2^	Δ*E*_1/2_^b^	*K*_c_^c^

**1a**	PF_6_^−^	606	700	94	39
	BArF_4_^−^	674	740	66	13
**1b**	PF_6_^−^	696	840	144	272
	BArF_4_^−^	784	891	107	64
**Ph1b**	PF_6_^−^	724	–	–	–

^a^In the presence of 0.1M *n*-Bu_4_NX. Potentials in mV vs. Fc*^+^/Fc* (Fc* = decamethylferrocene). ^b^Δ*E*_1/2_ = potential difference between two redox processes. ^c^Comproportionation constants obtained from *K*_c_ = exp(Δ*E*_1/2_ *F*/*RT*).

[1] $${\left(\text{1}\right)}_{2}\overset{E_{1/2}{}^{1}}{\underset{-e^{-}}{\to }}{\text{1}}^{+}\overset{E_{1/2}{}^{2+}}{\underset{-e^{-}}{\to }}{\text{1}}^{2+}$$

**Figure 3:**
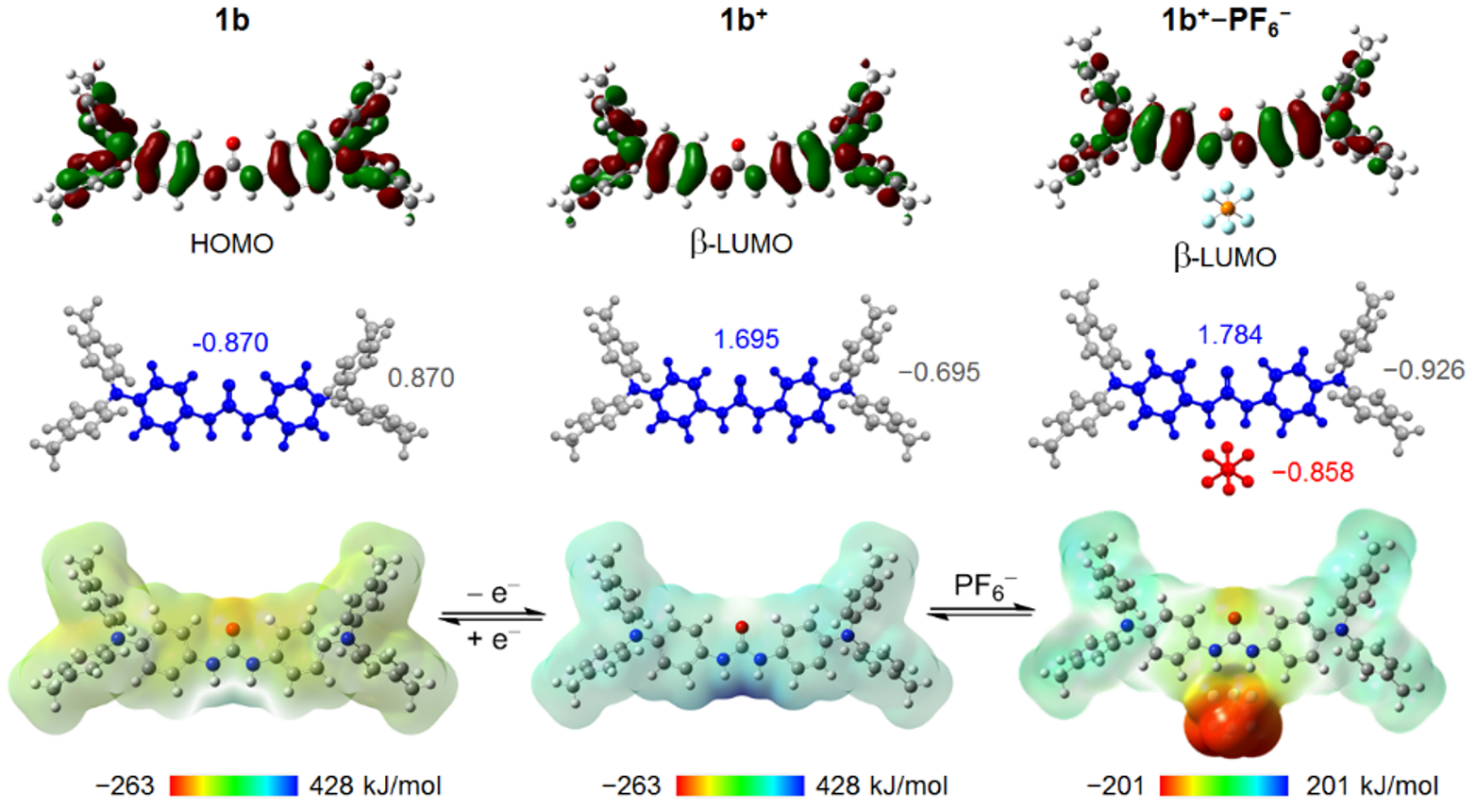
Key frontier orbitals (isosurface values 0.02 au) (top), DFT-optimized structures with Mullliken charges of peripheral tolyl groups (gray), nitrogen centers and bridging moieties (blue), and PF_6_^−^ (red) (middle), and electrostatic potential maps (isosurface value 0.0004 au) of **1b**, **1b^+^**, and **1b^+^**–PF_6_^–^ (bottom).

The highest occupied molecular orbitals (HOMOs) of the NAr_3_ components are hybridized, forming HOMO and HOMO−1 in both **1a** and **1b** as the antibonding and bonding combinations, respectively (Figure 3, top, and Supporting Information File 1, Figure S6). The differences between the HOMO and HOMO−1 energy levels were determined as 0.151 eV for **1a** and 0.155 eV for **1b**. The larger splitting for **1b** with respect to **1a** is a common feature between DFT calculations and electrochemical investigations. This indicates that electronic interactions between the NAr_3_ units largely contribute to the experimentally proven thermodynamic stability of the MV state. Indeed, the energy level differences in the neutral precursors have been taken as 2*H*_AB_ of the MV species in previous reports on radical cations of bis(NAr_3_) derivatives [11]. The HOMOs of **1a** and **1b** are distributed over both NAr_3_ units but not in the central carbonyl C=O moiety of the ureylene bridge. This means that the π orbital of the central C=O moiety does not contribute to the π-conjugation in the HOMOs. Such HOMO properties would be understood by comparison with a well-known reference bis(NAr_3_) derivative **MeO-TPD**, in which the NAr_3_ units are directly connected through a σ-bond [6]. **MeO-TPD** shows a larger energy difference between HOMO and HOMO−1 of 0.405 eV (Supporting Information File 1, Figure S7). In addition, the HOMO of **MeO-TPD** is delocalized over the whole molecule through the σ-bond bridge.

More interestingly, the supporting electrolytes were observed to cause unusual effects on the electrochemical behavior of **1b** (Figure 2, bottom). In the presence of *n*-Bu_4_NBArF_4_ (where ArF = 3,5-bis(trifluoromethyl)phenyl) as supporting electrolyte having a bulky counteranion, the potential splitting for **1b** became less pronounced and decreased by 37 mV compared to that with *n*-Bu_4_NPF_6_. A decreased potential splitting of 28 mV was also observed for **1a**. These observations corresponded to a 4.3-fold and 3.0-fold reduction in *K*_c_ for **1b** and **1a**, respectively, and implies that the larger counteranion (BArF_4_^−^) destabilized the MV state **1^+^**. This stands in contrast to general MV compounds were *K*_c_ values in the presence of larger counteranions increase because they do not form strong ion-pairs with charged species, enhancing electrostatic interactions between redox components [46–52]. Thus, a different mechanism is proposed to explain the present effects of the supporting electrolytes in terms of counteranion size, as discussed in the next section.

### Characterization of **1** in the MV state

DFT calculations were performed to obtain theoretical information about the MV state. The planarity of the urea moiety and the phenyl groups on both sides remained almost unchanged upon one-electron oxidation from **1** to **1^+^** (Supporting Information File 1, Figure S5). The enhanced acidity of the NH protons in **1b^+^**, compared to that in **1b**, was well predicted by electrostatic potential maps. The positive regions (blue) were found around the two hydrogen atoms of the urea bridge in the electrostatic potential maps of **1b^+^** (Figure 3, bottom). From these regions, **1b^+^** can form electrostatic interactions with negatively charged species. Indeed, an optimized structure of a complex of **1b^+^** and PF_6_^−^ was obtained and featured an intermolecular H-bonding between the N–H proton and the F atom of hexafluorophosphate. Such N–H···F hydrogen-bond formation was also reported for other urea derivatives with PF_6_^−^ as counteranion in the solid state [54–55]. The N···F distance of 2.85 Å in **1b^+^**–PF_6_^−^ is slightly longer than that observed in the crystal structure of a silver complex having a pyridyl urea ligand (2.67 and 2.75 Å), primarily reflecting the absence of packing in the former. In the optimized structure of **1a^+^**–PF_6_^−^, the N–H···F hydrogen-bonding was comparable to that in **1b^+^**–PF_6_^−^ with regard to the atomic geometry and the N···F distances.

The increased acidity of **1** upon one-electron-oxidation enhances the binding strength to PF_6_^−^, suggesting an involvement of the **1^+^**–PF_6_^−^ species during the electrochemical event of **1** described above. The comparison of DFT calculations between **1^+^** and **1^+^**–PF_6_^−^ revealed that upon zwitterion formation, the bound PF_6_^−^ can increase the electronic interactions between the NAr_3_ units. In the electrostatic potential maps of **1b^+^**–PF_6_^−^, the light-green and red regions face each other through the ureylene bridge, indicating the polarized nature of the zwitterionic species (Figure 3, bottom). The Mulliken charges of the central blue moiety involving redox-active nitrogen centers are 0.089 larger for **1b^+^**–PF_6_^−^ than for **1b^+^** (Figure 3, middle), meaning the larger positive charges are delocalized between the two nitrogen centers for **1b^+^**–PF_6_^−^ with the assistance of charge supply from the peripheral tolyl groups. In **1b^+^**–PF_6_^−^, the Mulliken positive charge distributions agree well with the distributions of β-LUMO (lowest unoccupied molecular orbital) (Figure 3, top). It should be noted that the β-LUMO of **1b^+^**–PF_6_^−^ is distributed in both the NAr_3_ units but not in the central C=O moiety of the ureylene bridge, which is in the same situation as the HOMO of **1a**. Upon binding with PF_6_^−^, the torsion of the central benzene rings in **1b^+^** decreased by 1.31°, accompanied with a slight shortening of the N···N distances (Supporting Information File 1, Figure S5). These structural features contribute to an increase in the electronic coupling between the NAr_3_ units, thereby increasing the potential splitting seen in the electrochemical measurement. This is consistent with previous reports on bis(NAr_3_)^•+^ derivatives that showed a positive correlation between the electron-richness of the bridge and the electronic coupling strength between the NAr_3_ units [8]. In contrast, **1b^+^** does not form a complex with the larger counteranion (BArF_4_^−^) because of steric hindrance but instead forms a conventional ion pair, resulting in a smaller potential splitting.

A polar solvent should interfere with the N–H···F H-bonding in the present MV state. Indeed, in MeCN/CH_2_Cl_2_ 9:1 containing *n*-Bu_4_NPF_6_, **1a** showed a 13 mV smaller potential splitting than in CH_2_Cl_2_ containing *n*-Bu_4_NPF_6_ (Supporting Information File 1, Figure S9 and Table S3). This suggests a decrease in electronic interactions between the NAr_3_ units upon disrupting the H-bonds. The mixed solvent was selected because of the low solubility of **1a** in MeCN. In general, in a more polar solvent, electrostatic repulsions between the redox units become smaller, thus decreasing potential splitting [46–48]. This is the case with **MeO-TPD** in CH_2_Cl_2_ and MeCN/CH_2_Cl_2_ 9:1 (Supporting Information File 1, Table S3). However, such electrostatic contribution to a change in the potential splitting should be smaller in case of compound **1a** because of the separated redox units (N···N 13.13 Å for **1a**^+^ in the DFT-optimized structure). Unfortunately, no investigations of solvent effects were performed with **1b** due to the low solubility in polar solvents including MeCN/CH_2_Cl_2_ mixed solvents.

**Figure 4:**
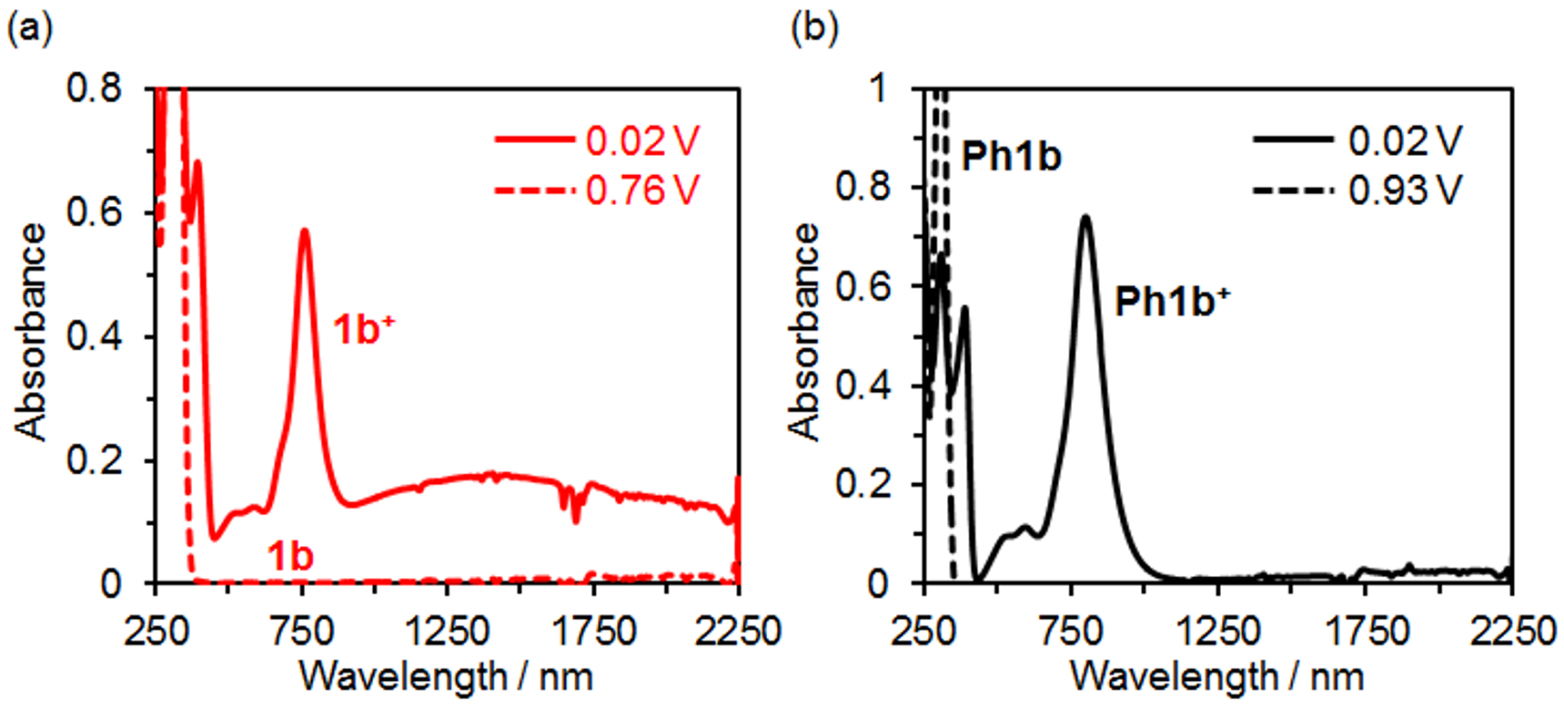
UV–vis-NIR spectral changes of CH_2_Cl_2_/*n*-Bu_4_NPF_6_ (0.10 M) solutions containing (a) **1b** (4.5 × 10^−4^ M) and (b) **Ph1b** (5.0 × 10^−4^ M) during the controlled potential electrolysis. Potentials in mV vs. Fc*^+^/Fc*.

To quantify the electronic interactions between the NAr_3_ units in the MV state, **1b** was investigated in CH_2_Cl_2_/*n*-Bu_4_NPF_6_ by a spectroelectrochemical method. The medium was chosen to reduce the influence of disproportionation to the **1b** and **1b^2+^** species. When the electrolysis of **1b** was performed at *E*_1/2_^1^, two new absorption bands were observed in this solution (Figure 4a). Based on its similarity to the reported NAr_3_^•+^ derivatives [56], the first band at 760 nm derives from the π–π* transition of the NAr_3_^•+^ moiety of **1b^+^** [56]. However, the second band in the NIR region significantly differs in broadness and intensity. Because any NIR absorption was not observed for the reference **Ph1b^+^** (Figure 4b), this second band of **1b^+^** is assigned to an IVCT transition from the NAr_3_ unit to the NAr_3_^•+^ unit. Indeed, using time-dependent DFT (TD-DFT) calculations, an electronic transition from the β-HOMO to β-LUMO was predicted in the NIR region (at 1745 nm) for **1b^+^** (Table S2 in Supporting Information File 1), which has an IVCT character corresponding to the experimentally observed one. The β-HOMO (194β) and β-LUMO (195β) for **1b^+^** are distributed over both NAr_3_ units (Figure 3 and Figure S6 in Supporting Information File 1). When the electrolysis is performed at *E*_1/2_^2^ + 0.15 V, the first band deriving from the π–π* transition of the NAr_3_^•+^ moiety increased in intensity (Supporting Information File 1, Figure S10), reflecting the presence of two chromophores in the generated two-electron-oxidized species **1b^2+^**. The generation of the dication agreed with the decrease in the IVCT intensity.

**Figure 5:**
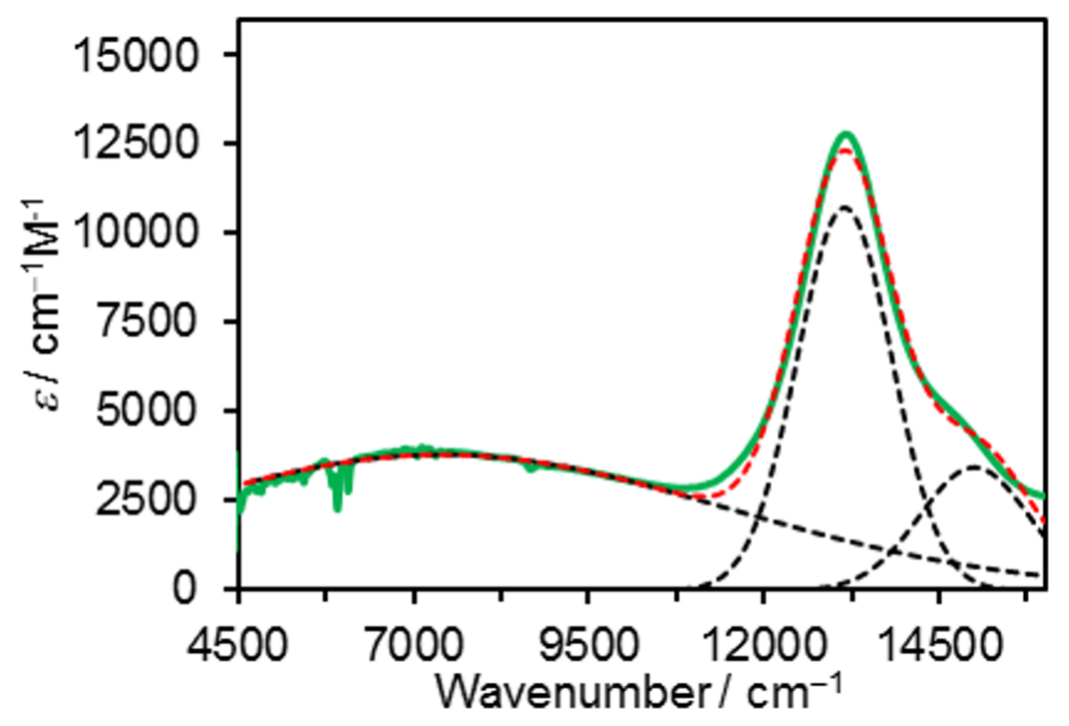
Vis-NIR spectra of **1b^+^** (green line) obtained by bulk electrolysis, with Gaussian deconvolutions (black broken lines) and the sum (red broken line).

The IVCT band of **1b^+^** was fitted using a Gaussian function (Figure 5) to obtain the spectroscopic parameters of energy (υ_max_), intensity (ε), and bandwidth at the half-height (Δυ_1/2_) (Table 2). An electronic coupling for **1b^+^**–PF_6_^−^ was calculated to be *H*_AB_ = 810 cm^−1^ using Hush analysis [1–5,19–20] with the three parameters. According to previous studies on bis(NAr_3_)^•+^ radical cations [6,8], we adopted the N···N distance of the DFT-optimized structure of **1b^+^**–PF_6_^−^ (13.12 Å) to determine *H*_AB_, although there is an uncertainty associated with the electron transfer distances in general organic MV systems [57–58]. As the IVCT bandwidth at half-height for **1b^+^** is broader than the high-temperature limit (47.94 × (Δυ_1/2_)^1/2^ = 4,120 cm^−1^) [6], **1b^+^** is regarded as a class II system.

**Table 2:** IVCT band shape and electronic coupling factor of **1^+^**.^a^

	υ_max_ (cm^−1^)	ε (M^−1^cm^−1^)	Δυ_1/2_ (cm^−1^)	*H*_AB_^b^ (cm^−1^)	α^c^

**1a^+^**	8550	3050	7590	700	0.082
**1b^+^**	7400	3810	9590	820	0.110
**FcFc^+ ^**^d^	8745	143	3323	165	0.019

^a^In CH_2_Cl_2_/0.1 M *n*-Bu_4_NPF_6_. ^b^Determined by the equation: *H*_AB_ = 0.0206 (υ_max_ ε_max_ Δυ_1/2_)^1/2^/*r*_DA_ (where *r*_DA_ is the N···N distance between NAr_3_ moieties). ^c^Delocalization parameter α = *H*_AB_/υ_max_. ^d^In CH_2_Cl_2_ with AgSbF_6_ as reported in reference [42].

The *H*_AB_ value for **1b^+^** is by a factor of 4.9 greater than that reported for its ferrocenyl counterpart (**FcFc^+^**) in the one-electron-oxidized form (*H*_AB_ = 165 cm^−1^) [42]. In conventional π-conjugated bis(NAr_3_) derivatives [8], it was clearly demonstrated that electron-rich bridges increased *H*_AB_ values. In the previous and present urea-bridged MV systems, the involved counteranions can enhance the electron-richness of the bridge moieties through interactions with NH protons. Such interactions can contribute to the relatively large *H*_AB_ values seen in **1b^+^** and **FcFc^+^**. The interaction parameter (α) is determined by the ratio of *H*_AB_ to λ and quantifies the degree of delocalization [1–5]. Changing the redox-active components from ferrocene to NAr_3_ led to a 4.3-fold increase in the α value. The degree of delocalization in the MV species can be understood in terms of the properties of the redox-active components; the positive charge of the ferrocenium moiety is accommodated largely on the d orbital, while that of the NAr_3_^•+^ moiety is delocalized from the nitrogen center to the benzene ring adjacent to the urea bridge.

The MV species **1a^+^** and the dication **1a^2+^** were also generated by the controlled-potential electrolysis and characterized by UV–vis-NIR spectroscopy (Figures S11, S12 in Supporting Information File 1 and Table 2). The replacement of the Me group at the *para* position by a OMe group decreased the *H*_AB_ values by 110 cm^−1^. Indeed, the Mulliken positive charges of the moieties covering the central bridge and nitrogen centers (blue regions) for **1a^+^**–PF_6_^−^ decreased by 0.142 compared to those for **1b^+^**–PF_6_^−^ (Figure S8 in Supporting Information File 1). This means that the peripheral electron-donating group of the NAr_3_ unit decreases the extent of delocalized positive charges. This is consistent with the previous reports on conventional π-conjugated bis(NAr_3_) derivatives [8]. In their pioneering work on polyacetylene-bridged bis(NAr_3_) derivatives, Lambert et al. demonstrated a negative linear correlation of ln (*H*_AB_) versus *n* − 1, where *n* is the bond number bridging the nitrogen centers of NAr_3_ moieties [6]. The *H*_AB_ value of **1a^+^** (*n* = 12) was almost comparable to that of a polyacetylene-bridged counterpart (*H*_AB_ = 710 cm^−1^ with *n* = 13). We found that the present urea bridge can maintain electronic coupling in terms of the N···N distances between the NAr_3_ moieties with a small decrease of *H*_AB_ values.

## Conclusion

A new series of urea-bridged bis(NAr_3_) derivatives was electrochemically characterized. This study represents the first example of ionic MV species whose thermodynamic stability was enhanced more by smaller counterions than by larger counterions. This was achieved by introduction of a urea bridge and subsequent H bonding with counteranions. The resultant zwitterionic MV species was well modeled by DFT calculations. Through the spectroelectrochemical method, we confirmed that the urea bridge can maintain electronic coupling between the NAr_3_ moieties in the MV cations. These findings provide new insights into controlling MV characteristics and fabricating sophisticated molecular devices through supramolecular methods.

## Experimental

### Materials and general measurements

All solvents and chemicals of reagent grade were used without purification except tetra-*n*-butylammonium phosphate (*n*-Bu_4_NPF_6_), which was recrystallized from methanol. 4-Aminotriphenylamine [59], 4,4’-dimethoxy-4’’-nitrotriphenylamine [38], 4,4’-dimethyl-4’’-nitrotriphenylamine [60], and *n*-Bu_4_NBArF_4_ [61] were synthesized as described in the literature. A JASCO V-670 spectrometer was used for UV–vis-NIR measurements at room temperature. The ^1^H NMR spectra were recorded using a JEOL JNM-ECP400 spectrometer with tetramethylsilane (TMS) as internal standard (0 ppm). EIMS measurements were performed using a JEOL JMS-700 MStation spectrometer.

### Synthesis and characterization of compounds

#### Synthesis of **1a**

The mixture of 4,4’-dimethoxy-4’’-nitrotriphenylamine (0.351 g, 1.00 mmol) and Pd/C (0.0145 g) was refluxed in dry ethanol (10 mL) for 1 h. After dropwise addition of hydrazine monohydrate (0.30 mL), the reaction mixture was refluxed overnight. After filtration of Pd/C and concentrating the filtrate to dryness, the resulting white solid (0.320 g) was identified as 4-amino-4’,4’’-dimethoxytriphenylamine by NMR comparison to reported data [62] which was used in the next step without further purification. To a solution of triphosgene (0.148 g, 0.500 mmol) in 15 mL of dry dichloromethane a solution of the crude product of 4-amino-4’,4’’-dimethoxytriphenylamine (0.32 g) and 0.28 mL of triethylamine in dry dichloromethane (15 mL) was added at 0 °C. After 10 min of stirring, 2.5 mL of dry pyridine were added and the mixture heated at 50 °C overnight. The reaction mixture was filtrated and concentrated to dryness. The resulting solid was dissolved in ethyl acetate and washed with water. After drying the organic layer over Na_2_SO_4_, the solution was concentrated to dryness. Compound **1a** was isolated by column chromatography on silica gel using ethyl acetate/*n*-hexane 1:1 as the eluent. Yield: 0.043 g (13%). ^1^H NMR (400 MHz, DMSO-*d*_6_, ppm) δ 3.71 (s, 12H, -C*H*_3_), 6.80 (m, 4H, -NCC(*H*)C(H)CN(H)-), 6.85, 6.90 (m, 16H, -OCC(*H*)C(*H*)CN-), 7.28 (m, 4H, -NCC(H)C(*H*)CN(H)-), 8.44 (s, 2H, -N*H*-); HREIMS (*m*/*z*): [M + Na]^+^ calcd for C_41_H_38_N_4_NaO_5_, 689.27399; found: 689.27404.

#### Synthesis of **1b**

Following a similar procedure as described for **1a** and starting from 4,4’-dimethyl-4’’-nitrotriphenylamine (0.637 g, 2.00 mmol), the target compound was synthesized and purified. Yield: 0.165 g (27%). ^1^H NMR (400 MHz, DMSO-*d*_6_, ppm) δ 2.23 (s, 12H, -C*H*_3_), 6.83 (m, 8H), 6.89 (m, 4H, -NCC(*H*)C(H)CN(H)-), 7.05 (m, 8H), 7.34 (m, 4H, -NCC(H)C(*H*)CN(H)-), 8.54 (s, 2H, -N*H*-); HREIMS (*m*/*z*): [M + Na]^+^ calcd for C_41_H_38_N_4_NaO, 625.29433; found: 625.29533.

#### Synthesis of **1c**

To a solution of triphosgene (0.296 g, 1.00 mmol) in 15 mL of dry dichloromethane a solution of the crude product of 4-aminotriphenylamine (0.520 g, 2.00 mmol) and 0.70 mL of triethylamine in dry dichloromethane (15 mL) was added at 0 °C. The reaction mixture was heated at 50 °C overnight and filtered. The filtrate was concentrated to dryness, the resulting solid was dissolved in chloroform and washed with water. After drying the organic layer over Na_2_SO_4_, the solution was concentrated to afford the target product **1c**. Yield: 0.231 g (42%). ^1^H NMR (400 MHz, DMSO-*d*_6_, ppm) δ 6.96 (m, 16H, -C(*H*)C(H)C(*H*)N-, -NC(*H*)C(H)CN(H)-), 7.25 (m, 8H, -C(H)C(*H*)C(H)N-), 7.41 (m, 4H, -NC(H)C(*H*)CN(H)-), 8.63 (s, 2H, -N*H*-); HREIMS (*m*/*z*): [M + Na]^+^ calcd for C_37_H_30_N_4_NaO, 569.23173; found: 569.23174.

#### Synthesis of **Ph1b**

The mixture of 4,4’-dimethyl-4”-nitrotriphenylamine (0.318 g, 1.00 mmol) and Pd/C (0.0145 g) in dry ethanol (10 mL) was refluxed for 1 h. After dropwise addition of hydrazine monohydrate (0.30 mL), the reaction mixture was refluxed overnight. After filtration and concentrating the filtrate to dryness, the resulting white solid (0.320 g) was identified as 4-amino-4’,4’’-dimethyltriphenylamine by NMR [62] and used in the next step without further purification. To a solution of triphosgene (0.296 g, 1.00 mmol) in 15 mL of dry dichloromethane at 0 °C a solution of aniline (0.093 g, 1.00 mmol) and 0.56 mL of triethylamine in dry dichloromethane (15 mL) was added. After 10 min of stirring, 5.0 mL of dry pyridine were added followed by crude product of 4-amino-4’,4’’-dimethyltriphenylamine (0.288 g). The resulting reaction mixture was heated at 50 °C overnight. After filtration the filtrate was concentrated, diluted with dichloromethane and washed with water. After drying the organic layer over Na_2_SO_4_, the solution was concentrated to dryness. Compound **Ph1b** was isolated by column chromatography on silica gel using ethyl acetate/*n*-hexane 1:1. Yield: 0.086 g (21%). ^1^H NMR (400 MHz, DMSO-*d*_6_, ppm) δ 2.23 (s, 6H, -C*H*_3_), 6.83 (m, 4H, -NCC(*H*)C(*H*)C(CH_3_)-), 6.90 (m, 2H, -N(H)CC(H)C(*H*)CN-), 6.94 (m, 1H, -C(*H*)C(H)C(H)CN-), 7.05 (m, 4H, -NCC(*H*)C(*H*)C(CH_3_)-), 7.26 (m, 2H, -C(H)C(*H*)C(H)CN-), 7.35, 7.43 (m, 4H, -C(H)C(H)C(*H*)CN-, -N(H)CC(*H*)C(H)CN-), 8.58, 8.59 (s, 2H, -N*H*-); HREIMS (*m*/*z*): [M + Na]^+^ calcd for C_27_H_25_N_3_NaO, 430.18953; found: 430.18889.

#### DFT calculations

The DFT calculations were performed using the Gaussian09 software [63]. The three-parameterized Becke–Lee–Yang–Parr (B3LYP) hybrid exchange-correlation functional [64] was selected using the 6-31G(d) basis set for **1a** and **1b** with a restricted method and using the 6-311++G(d,p) basis set for the MV species with an unrestricted method. Vibrational frequencies were calculated to check the stability of the optimized structures and confirm that there are no imaginary frequencies. The TD-DFT calculations were also performed to predict electronic transitions with energies and oscillator strengths to obtain insight into UV–vis-NIR spectral data.

#### Electrochemical investigations

The electrochemical behavior was investigated using a BAS electrochemical analyzer (Bioanalytical Systems Inc, West Lafayette, IN, USA) with a three-electrode system composed of a platinum wire (1.6 mm diameter) counter electrode, a glassy carbon working electrode (3.0 mm diameter), and a Ag/AgCl (3.0 M NaCl) reference electrode in CH_2_Cl_2_ solutions (1.0 mM) of the target compound containing 0.1 M *n*-Bu_4_NPF_6_. Additional experiments were carried out in the presence of decamethylferrocene (Fc*). The potentials versus the Fc^+^/Fc couple (where Fc = ferrocene) are also included in Table S4 (Supporting Information File 1), which are based on an independent experiment containing Fc and Fc*.

## Supporting Information

File 1 Copies of ^1^H NMR spectra of new compounds, DFT calculation data, and electrochemical data.

## References

[1] Demadis K D, Hartshorn C M, Meyer T J (2001). Chem Rev.

[2] Brunschwig B S, Creutz C, Sutin N (2002). Chem Soc Rev.

[3] D’Alessandro D M, Keene F R (2006). Chem Soc Rev.

[4] D'Alessandro D M, Keene F R (2006). Chem Rev.

[5] Aguirre-Etcheverry P, O’Hare D (2010). Chem Rev.

[6] Lambert C, Nöll G (1999). J Am Chem Soc.

[7] Heckmann A, Amthor S, Lambert C (2006). Chem Commun.

[8] Barlow S, Risko C, Odom S A, Zheng S, Coropceanu V, Beverina L, Brédas J-L, Marder S R (2012). J Am Chem Soc.

[9] Low P J, Paterson M A J, Puschmann H, Goeta A E, Howard J A K, Lambert C, Cherryman J C, Tackley D R, Leeming S, Brown B (2004). Chem – Eur J.

[10] Uebe M, Kazama T, Kurata R, Sakamaki D, Ito A (2017). Angew Chem, Int Ed.

[11] Lambert C, Risko C, Coropceanu V, Schelter J, Amthor S, Gruhn N E, Durivage J C, Brédas J-L (2005). J Am Chem Soc.

[12] Reuter L G, Bonn A G, Stückl A C, He B, Pati P B, Zade S S, Wenger O S (2012). J Phys Chem A.

[13] Schmidt H C, Spulber M, Neuburger M, Palivan C G, Meuwly M, Wenger O S (2016). J Org Chem.

[14] Shen J-J, Shao J-Y, Zhu X, Zhong Y-W (2016). Org Lett.

[15] Burrezo P M, Lin N-T, Nakabayashi K, Ohkoshi S-i, Calzado E M, Boj P G, Díaz García M A, Franco C, Rovira C, Veciana J (2017). Angew Chem, Int Ed.

[16] Schäfer J, Holzapfel M, Mladenova B, Kattnig D, Krummenacher I, Braunschweig H, Grampp G, Lambert C (2017). J Am Chem Soc.

[17] Zhang Y-M, Wu S-H, Yao C-J, Nie H-J, Zhong Y-W (2012). Inorg Chem.

[18] Tahara K, Koyama H, Fujitsuka M, Tokunaga K, Lei X, Majima T, Kikuchi J-I, Ozawa Y, Abe M (2019). J Org Chem.

[19] Hankache J, Wenger O S (2011). Chem Rev.

[20] Heckmann A, Lambert C (2012). Angew Chem, Int Ed.

[21] He B, Wenger O S (2011). J Am Chem Soc.

[22] Wenger O S (2012). Chem Soc Rev.

[23] Cooke G, Rotello V M (2002). Chem Soc Rev.

[24] Bu J, Lilienthal N D, Woods J E, Nohrden C E, Hoang K T, Truong D, Smith D K (2005). J Am Chem Soc.

[25] Woods J E, Ge Y, Smith D K (2008). J Am Chem Soc.

[26] Clare J P, Statnikov A, Lynch V, Sargent A L, Sibert J W (2009). J Org Chem.

[27] Amendola V, Fabbrizzi L, Mosca L (2010). Chem Soc Rev.

[28] Li A-F, Wang J-H, Wang F, Jiang Y-B (2010). Chem Soc Rev.

[29] Dydio P, Lichosyt D, Jurczak J (2011). Chem Soc Rev.

[30] Li Y, Park T, Quansah J K, Zimmerman S C (2011). J Am Chem Soc.

[31] Clare L A, Pham A T, Magdaleno F, Acosta J, Woods J E, Cooksy A L, Smith D K (2013). J Am Chem Soc.

[32] Tamashiro B T, Cedano M R, Pham A T, Smith D K (2015). J Phys Chem C.

[33] Clare L A, Smith D K (2016). Chem Commun.

[34] Cedano M R, Smith D K (2018). J Org Chem.

[35] Tadokoro M, Inoue T, Tamaki S, Fujii K, Isogai K, Nakazawa H, Takeda S, Isobe K, Koga N, Ichimura A (2007). Angew Chem, Int Ed.

[36] Wilkinson L A, McNeill L, Meijer A J H M, Patmore N J (2013). J Am Chem Soc.

[37] Wilkinson L A, McNeill L, Scattergood P A, Patmore N J (2013). Inorg Chem.

[38] Tahara K, Nakakita T, Katao S, Kikuchi J-i (2014). Chem Commun.

[39] Jin-Long, Matsuda Y, Uemura K, Ebihara M (2015). Inorg Chem.

[40] Tadokoro M, Hosoda H, Inoue T, Murayama A, Noguchi K, Iioka A, Nishimura R, Itoh M, Sugaya T, Kamebuchi H (2017). Inorg Chem.

[41] Mahmoud K, Long Y-T, Schatte G, Kraatz H-B (2004). J Organomet Chem.

[42] Siebler D, Förster C, Gasi T, Heinze K (2011). Organometallics.

[43] Gong Z-L, Deng L-Y, Zhong Y-W, Yao J (2017). Phys Chem Chem Phys.

[44] Gong Z-L, Zhong Y-W, Yao J (2015). Chem – Eur J.

[45] Corbin P S, Zimmerman S C, Thiessen P A, Hawryluk N A, Murray T J (2001). J Am Chem Soc.

[46] Hildebrandt A, Lang H (2013). Organometallics.

[47] Winter R F (2014). Organometallics.

[48] Diallo A K, Absalon C, Ruiz J, Astruc D (2011). J Am Chem Soc.

[49] Tahara K, Terashita N, Akita T, Katao S, Kikuchi J-i, Tokunaga K (2015). Organometallics.

[50] Hildebrandt A, Miesel D, Lang H (2018). Coord Chem Rev.

[51] Tahara K, Akita T, Katao S, Tokunaga K, Kikuchi J-i (2014). Dalton Trans.

[52] Tahara K, Akita T, Katao S, Kikuchi J-i (2014). Dalton Trans.

[53] Bender T P, Graham J F, Duff J M (2001). Chem Mater.

[54] Custelcean R (2008). Chem Commun.

[55] Blondeau P, van der Lee A, Barboiu M (2005). Inorg Chem.

[56] Sreenath K, Suneesh C V, Ratheesh Kumar V K, Gopidas K R (2008). J Org Chem.

[57] Brunschwig B S, Creutz C, Sutin N (1998). Coord Chem Rev.

[58] Kattnig D R, Mladenova B, Grampp G, Kaiser C, Heckmann A, Lambert C (2009). J Phys Chem C.

[59] Lee W-Y, Kurosawa T, Lin S-T, Higashihara T, Ueda M, Chen W-C (2011). Chem Mater.

[60] Liu X, Zhang S (2011). Synlett.

[61] Barrière F, Geiger W E (2006). J Am Chem Soc.

[62] Moulin E, Niess F, Maaloum M, Buhler E, Nyrkova I, Giuseppone N (2010). Angew Chem, Int Ed.

[63] (2009). Gaussian 09.

[64] Becke A D (1993). J Chem Phys.

