# Supplementary material for: Small anion-assisted electrochemical potential splitting in a new series of bistriarylamine derivatives: organic mixed valency across a urea bridge and zwitterionization
Source: Beilstein J Org Chem. 2019 Sep 24;15:2277–86. doi: 10.3762/bjoc.15.220 (PMC6774064; doi:10.3762/bjoc.15.220)
Supplement: File 1 — Copies of 1H NMR spectra of new compounds, DFT calculation data, and electrochemical data. [file Beilstein_J_Org_Chem-15-2277-s001.pdf]

BEILSTEIN JOURNAL OF ORGANIC CHEMISTRY

Supporting Information

for

Small anion-assisted electrochemical potential splitting in a new series of bistriarylamine derivatives: organic mixed valency across a urea bridge and zwitterionization

Keishiro Tahara, Tetsufumi Nakakita, Alyona A. Starikova, Takashi Ikeda, Masaaki Abe and Jun-ichi Kikuchi

Beilstein J. Org. Chem. **2019,** *15,* 2277–2286. doi:10.3762/bjoc.15.220

Copies of ^{1}H NMR spectra of new compounds, DFT calculation data, and electrochemical data

License and Terms: This is a supporting information file under the terms of the Creative Commons Attribution License (http://creativecommons.org/licenses/by/4.0). Please note that the reuse, redistribution and reproduction in particular requires that the authors and source are credited.
The license is subject to the *Beilstein Journal of Organic Chemistry* terms and conditions: (https://www.beilstein-journals.org/bjoc)

Contents

1. Characterization of new compounds

2. DFT calculation

3. Electrochemical investigation

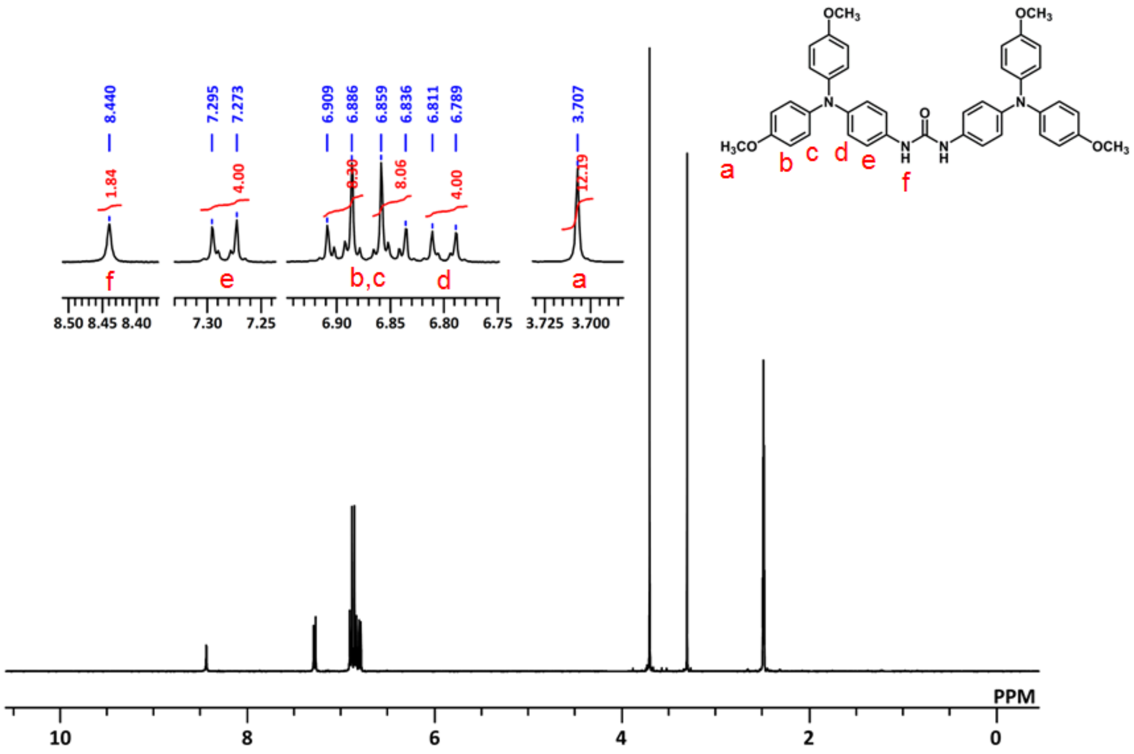

Figure S1: ^{1}H NMR spectrum of **1a** in DMSO-d_6 (400 MHz).

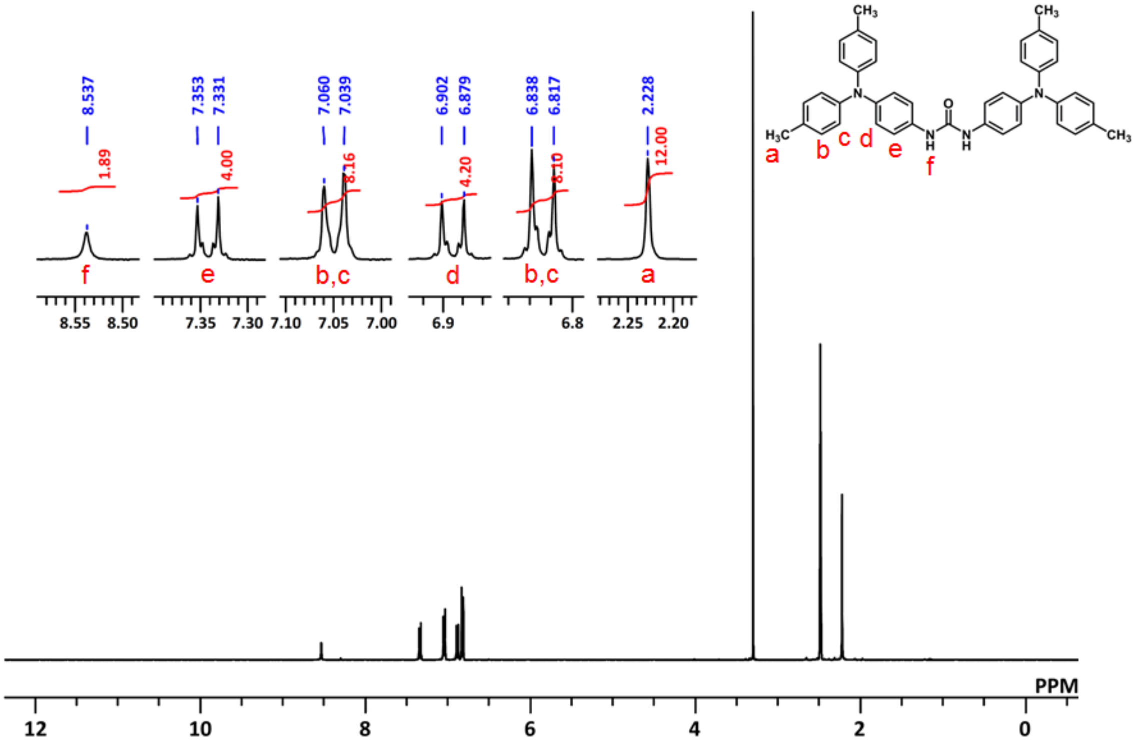

Figure S2: ^{1}H NMR spectrum of **1b** in DMSO-d_6 (400 MHz).

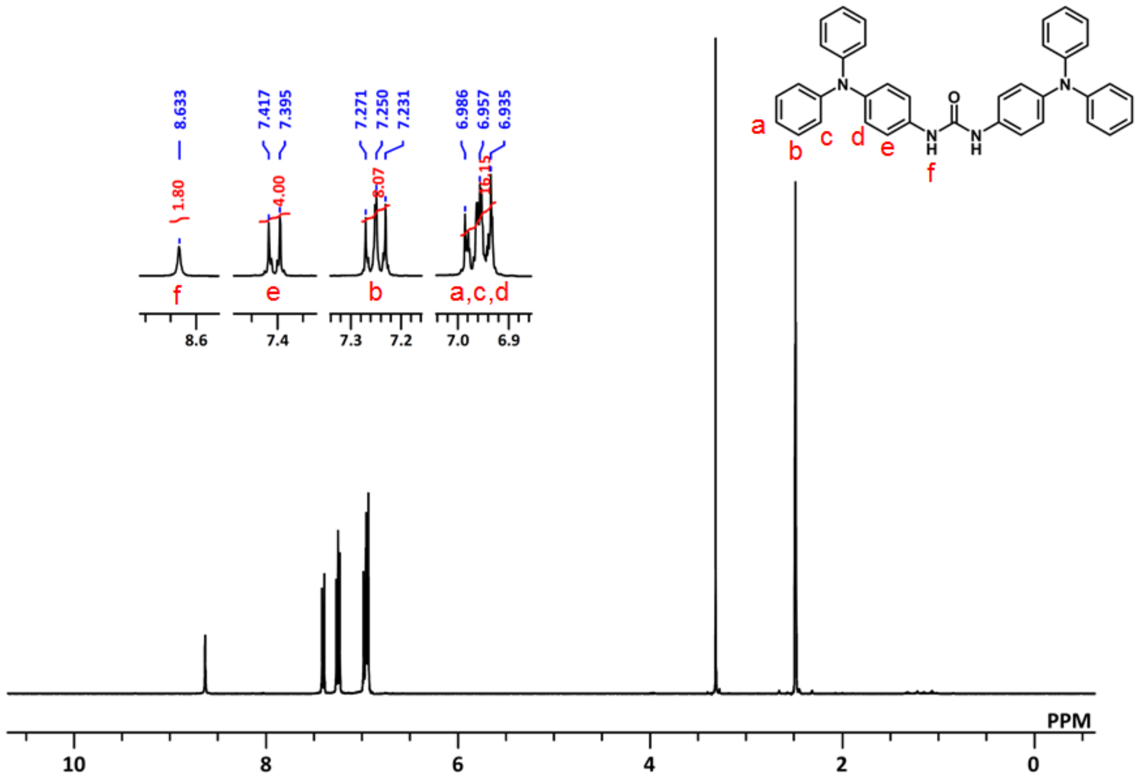

Figure S3: ^{1}H NMR spectrum of **Ph1b** in DMSO-d_6 (400 MHz).

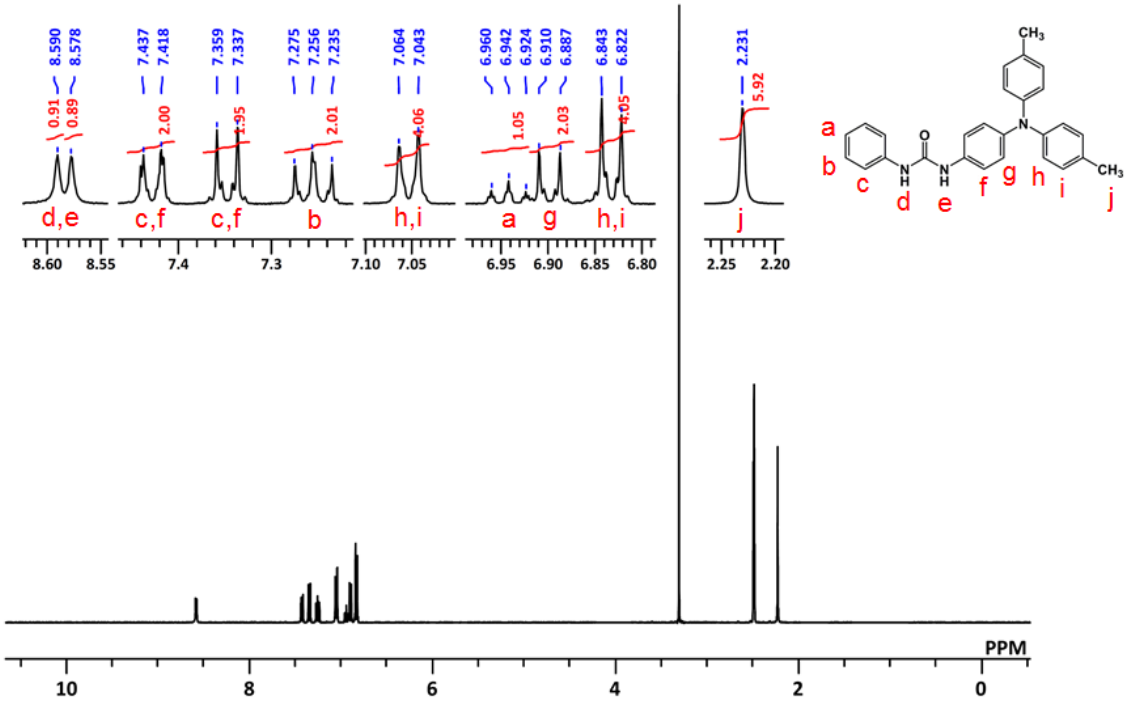

Figure S4: ^{1}H NMR spectrum of **1c** in DMSO-d_6 (400 MHz).

2. DFT calculation

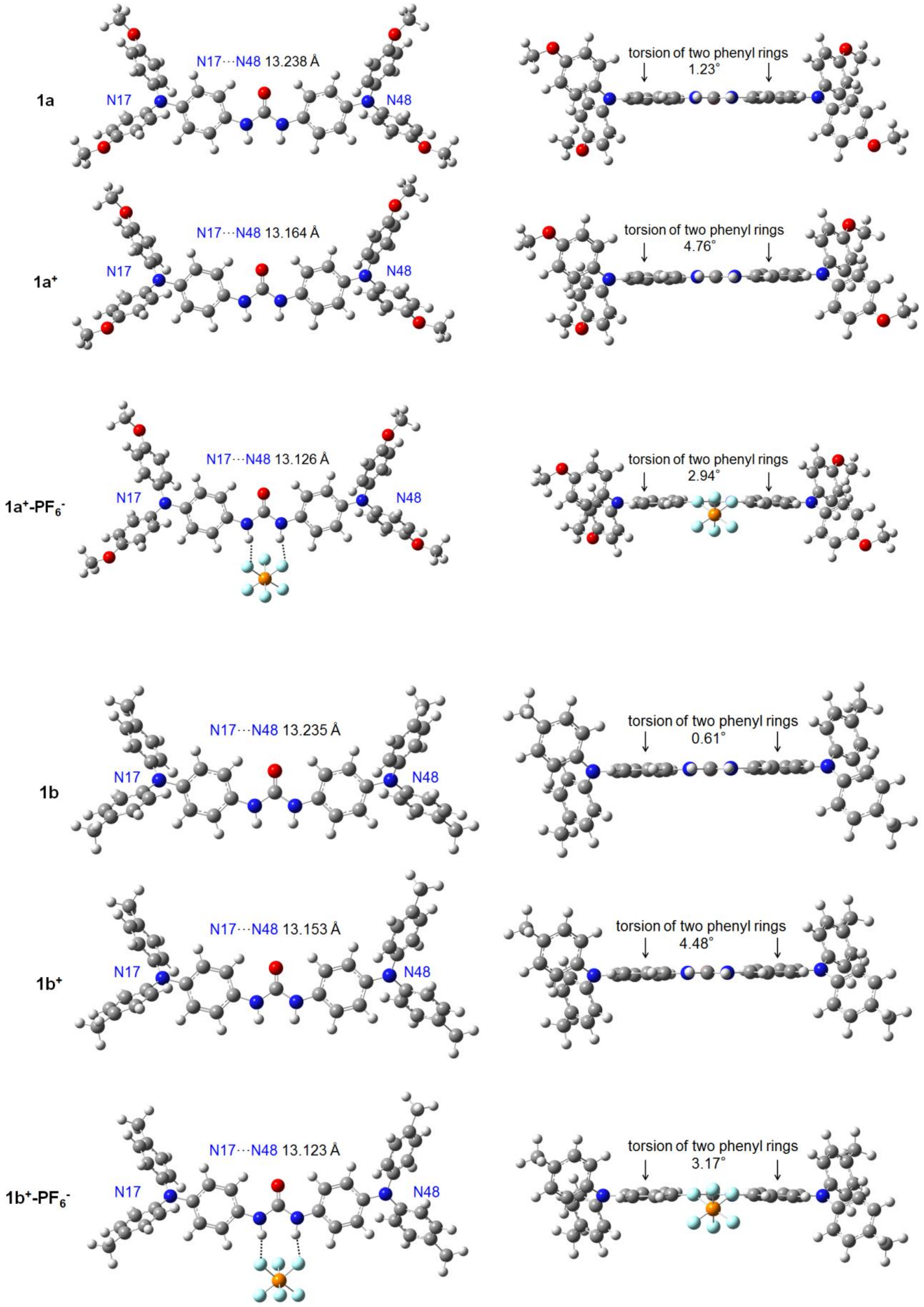

Figure S5: DFT-optimized structures of (top) **1a** and (bottom) **1b** and those in the MV state.

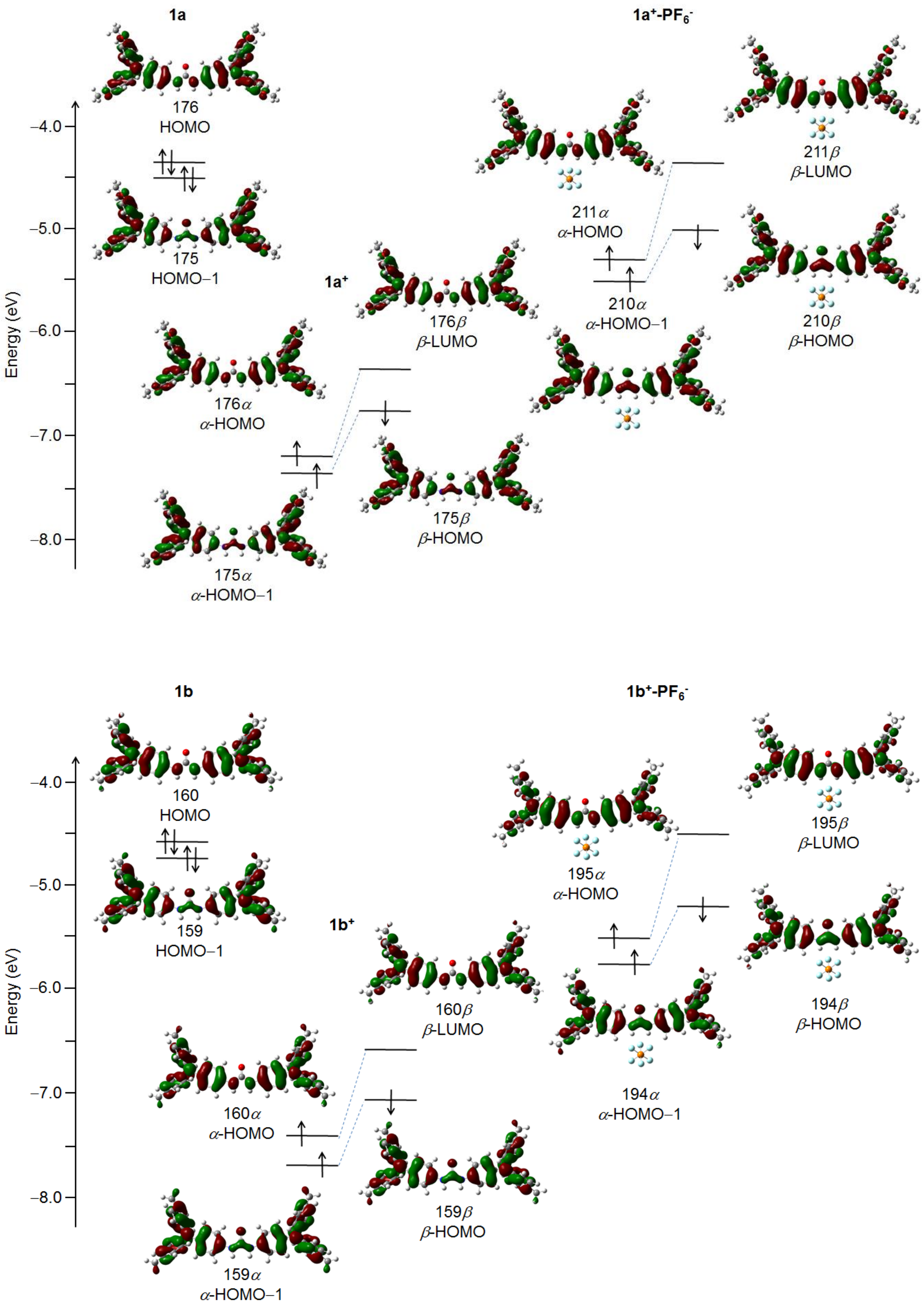

Figure S6: Selected molecular orbitals of (top) **1a** and (bottom) **1b** and those in the MV state.

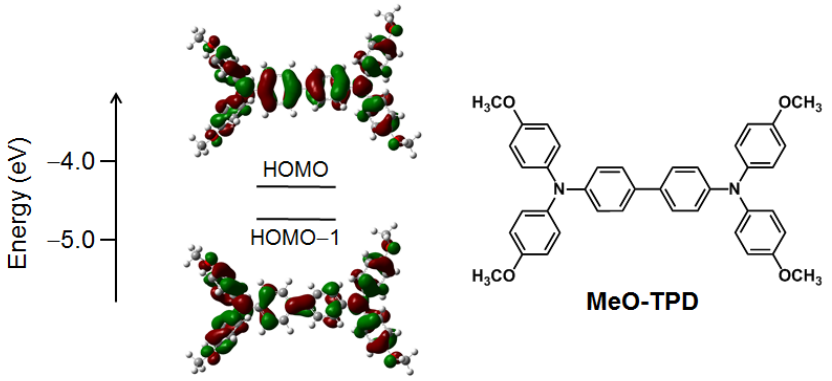

Figure S7: Chemical structure of **MeO-TPD** and its selected molecular orbitals.

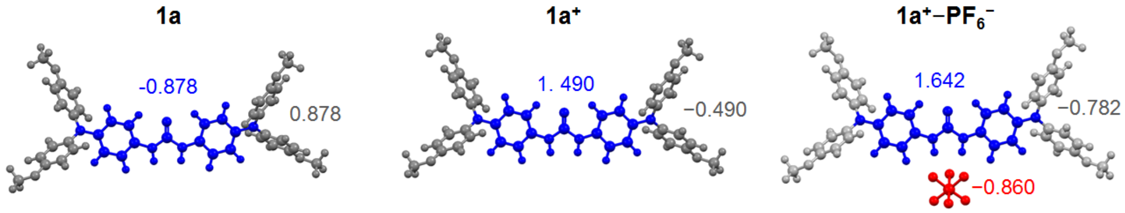

Figure S8: DFT-optimized structures with Mullliken charges of peripheral anisyl groups (gray), nitrogen centers and bridging moieties (blue), and PF_6^- (red).

Table S1: Coordinates of optimized gas-phase geometries of urea derivatives.

1a with the 6-31G(d) basis set

Center Number	Atomic Number	Coordinates (Å)		
		X	Y	Z
1	8	0.000015	0.444912	-0.000003
2	7	1.151099	-1.554559	0.019771
3	7	-1.15114	-1.554514	-0.01933
4	6	-0.000006	-0.779383	0.000155
5	1	1.040931	-2.558974	0.004804
6	1	-1.041014	-2.558931	-0.004196
7	6	-2.494951	-1.123456	-0.048043
8	6	-3.488013	-2.115328	-0.049695
9	6	-2.890872	0.222443	-0.075858
10	6	-4.836603	-1.783705	-0.087178
11	1	-3.202102	-3.166183	-0.036366
12	1	-2.140016	0.999483	-0.067753
13	6	-4.244581	0.547852	-0.09129
14	6	-5.242264	-0.439494	-0.102
15	1	-5.583179	-2.57104	-0.101486
16	1	-4.533434	1.594055	-0.097566
17	7	-6.617943	-0.094431	-0.127355
18	6	-7.065973	1.005654	-0.910913
19	6	-7.554798	-0.841899	0.638925
20	6	-6.600247	1.191707	-2.225294
21	6	-7.98604	1.92658	-0.396886
22	6	-8.785877	-1.226269	0.094805

23	6	-7.270236	-1.207843	1.967396
24	1	-5.889475	0.48603	-2.643799
25	6	-7.032644	2.267973	-2.987121
26	6	-8.443957	2.998381	-1.166436
27	1	-8.35432	1.802034	0.616657
28	1	-9.022734	-0.954013	-0.929016
29	6	-9.717587	-1.944498	0.847838
30	6	-8.181534	-1.941516	2.713616
31	1	-6.323684	-0.913378	2.409665
32	1	-6.671724	2.415004	-4.000428
33	6	-7.962878	3.179951	-2.467484
34	1	-9.159918	3.687782	-0.733281
35	1	-10.662019	-2.219704	0.391815
36	6	-9.416704	-2.313671	2.163292
37	1	-7.963366	-2.225599	3.738554
38	6	2.494932	-1.123556	0.048376
39	6	2.890922	0.222323	0.076041
40	6	3.487944	-2.115484	0.05011
41	6	4.244652	0.547663	0.091401
42	1	2.140109	0.999404	0.067873
43	6	4.836548	-1.783927	0.087529
44	1	3.201969	-3.166324	0.036908
45	6	5.242279	-0.439732	0.102181
46	1	4.533564	1.593851	0.097578
47	1	5.583093	-2.57129	0.101916
48	7	6.617979	-0.094751	0.127535
49	6	7.554782	-0.842074	-0.63894
50	6	7.065983	1.005401	0.91102
51	6	7.269964	-1.208178	-1.967322
52	6	8.786075	-1.226152	-0.09511
53	6	7.985824	1.926473	0.396891
54	6	6.600405	1.191354	2.225478
55	6	8.181224	-1.941707	-2.713718
56	1	6.323246	-0.913923	-2.409375
57	6	9.717753	-1.944244	-0.848324
58	1	9.023126	-0.953796	0.928639
59	1	8.35398	1.802016	-0.616708
60	6	8.443712	2.998329	1.166407
61	6	7.032762	2.267642	2.987273
62	1	5.889785	0.485558	2.644042
63	6	9.416627	-2.31356	-2.16368
64	1	7.962881	-2.225914	-3.738583
65	1	10.66236	-2.219236	-0.39254
66	1	9.159459	3.687873	0.733128
67	6	7.962807	3.179786	2.467527
68	1	6.671997	2.414622	4.000642
69	8	-8.332794	4.197677	-3.303791
70	8	-10.246333	-3.025323	2.986576
71	6	-9.257861	5.154739	-2.818154
72	1	-8.867712	5.685547	-1.939132
73	1	-9.408225	5.867547	-3.631317
74	1	-10.220616	4.693143	-2.55923
75	6	-11.503255	-3.432426	2.474954
76	1	-11.391885	-4.096387	1.606839

77	1	-11.995066	-3.977183	3.283244
78	1	-12.123392	-2.57166	2.189445
79	8	8.332695	4.19749	3.303835
80	8	10.246187	-3.025122	-2.987087
81	6	9.258033	5.154362	2.818419
82	1	8.867958	5.68562	1.939629
83	1	9.408791	5.866864	3.63178
84	1	10.220571	4.692489	2.559168
85	6	11.50365	-3.431178	-2.476025
86	1	11.393192	-4.095295	-1.607887
87	1	11.995606	-3.975483	-3.28453
88	1	12.123153	-2.5699	-2.190686

1a^{+} with the 6-311++G(d,p) basis set

Center Number	Atomic Number	Coordinates (Å)		
		X	Y	Z
1	8	-0.000018	0.421377	-0.000548
2	7	-1.151882	-1.571501	-0.000461
3	7	1.151951	-1.57144	-0.00053
4	6	0.000014	-0.793894	-0.000522
5	1	-1.04071	-2.573831	0.040025
6	1	1.040834	-2.573779	-0.040918
7	6	2.484782	-1.138603	0.024813
8	6	3.483561	-2.1288	-0.007899
9	6	2.87349	0.210455	0.08441
10	6	4.823389	-1.794195	0.019655
11	1	3.202909	-3.177273	-0.039723
12	1	2.122467	0.984309	0.093175
13	6	4.218189	0.54151	0.109886
14	6	5.220336	-0.444618	0.079745
15	1	5.571461	-2.576264	0.009973
16	1	4.500646	1.586096	0.139893
17	7	6.581212	-0.095614	0.111745
18	6	7.017687	1.032581	0.857597
19	6	7.537168	-0.866345	-0.604492
20	6	6.529094	1.266463	2.155558
21	6	7.951613	1.92313	0.317222
22	6	8.753871	-1.219036	-0.01119
23	6	7.282014	-1.274675	-1.925643
24	1	5.819275	0.57607	2.594525
25	6	6.957558	2.36212	2.879844
26	6	8.392418	3.024062	1.044225
27	1	8.331493	1.758404	-0.683634
28	1	8.96043	-0.913887	1.007325
29	6	9.699625	-1.963006	-0.709327
30	6	8.21463	-2.019257	-2.622168
31	1	6.352588	-0.99117	-2.404292
32	1	6.594062	2.544148	3.883639
33	6	7.894325	3.254782	2.333047
34	1	9.108587	3.697999	0.594557
35	1	10.627326	-2.226031	-0.220043
36	6	9.43447	-2.373246	-2.022288
37	1	8.031972	-2.328034	-3.64405

38	6	-2.484737	-1.138733	-0.025653
39	6	-2.873523	0.210295	-0.085405
40	6	-3.483465	-2.128974	0.007392
41	6	-4.218242	0.541284	-0.110652
42	1	-2.12254	0.984184	-0.094445
43	6	-4.823313	-1.794435	-0.019932
44	1	-3.202758	-3.177429	0.039312
45	6	-5.22034	-0.444882	-0.080113
46	1	-4.500756	1.585852	-0.140779
47	1	-5.571344	-2.576538	-0.00999
48	7	-6.581228	-0.095933	-0.111847
49	6	-7.537042	-0.866489	0.604718
50	6	-7.017831	1.032227	-0.857734
51	6	-7.281442	-1.275029	1.925728
52	6	-8.754112	-1.218796	0.011918
53	6	-7.951232	1.923128	-0.317063
54	6	-6.529851	1.265666	-2.155993
55	6	-8.213957	-2.019428	2.622579
56	1	-6.351745	-0.991817	2.404022
57	6	-9.699769	-1.962564	0.710394
58	1	-8.961034	-0.913503	-1.006478
59	1	-8.330632	1.758723	0.684029
60	6	-8.392136	3.024009	-1.044091
61	6	-6.958421	2.361261	-2.880318
62	1	-5.820427	0.574988	-2.595153
63	6	-9.434162	-2.373012	2.023199
64	1	-8.030941	-2.328354	3.644351
65	1	-10.627756	-2.22528	0.221487
66	1	-9.107894	3.698236	-0.594203
67	6	-7.894669	3.254287	-2.333233
68	1	-6.595404	2.542963	-3.884346
69	8	8.250191	4.293148	3.123726
70	8	10.278281	-3.10127	-2.789408
71	6	9.202538	5.240055	2.640393
72	1	8.833962	5.747398	1.74363
73	1	9.328166	5.965122	3.44145
74	1	10.162937	4.760245	2.428534
75	6	11.541039	-3.495713	-2.253582
76	1	11.414298	-4.137878	-1.376621
77	1	12.033432	-4.056344	-3.045079
78	1	12.148691	-2.624057	-1.991894
79	8	-8.250698	4.292555	-3.123973
80	8	-10.277846	-3.100865	2.79062
81	6	-9.202556	5.239808	-2.640359
82	1	-8.833371	5.74729	-1.743924
83	1	-9.328451	5.964705	-3.441528
84	1	-10.162954	4.76028	-2.427854
85	6	-11.540993	-3.49481	2.255344
86	1	-11.414891	-4.13694	1.378265
87	1	-12.033218	-4.055337	3.047018
88	1	-12.148451	-2.62291	1.994018

1a^{+}-PF_6^{-} with the 6-311++G(d,p) basis set

Center Number	Atomic Number	Coordinates (Å)		
		X	Y	Z
1	8	0.001848	1.340148	0.143598
2	7	1.140555	-0.663576	0.266274
3	7	-1.147761	-0.658312	0.248675
4	6	-0.001429	0.120834	0.21162
5	1	1.026178	-1.676588	0.308231
6	1	-1.038772	-1.671315	0.303557
7	6	-2.463613	-0.230157	0.177298
8	6	-3.461892	-1.231669	0.183173
9	6	-2.861758	1.121035	0.099707
10	6	-4.796802	-0.899969	0.112771
11	1	-3.164317	-2.273011	0.224287
12	1	-2.111458	1.895778	0.107801
13	6	-4.204173	1.444262	0.030393
14	6	-5.202082	0.449584	0.033394
15	1	-5.540102	-1.686484	0.099348
16	1	-4.492319	2.486882	-0.013095
17	7	-6.560203	0.788257	-0.041886
18	6	-6.982177	1.943797	-0.758768
19	6	-7.543906	-0.022258	0.594758
20	6	-6.477849	2.214576	-2.041552
21	6	-7.918777	2.819992	-0.206073
22	6	-8.705677	-0.395011	-0.084378
23	6	-7.36759	-0.451415	1.920284
24	1	-5.760236	1.537152	-2.488175
25	6	-6.893619	3.334708	-2.739291
26	6	-8.350162	3.943845	-0.907846
27	1	-8.315056	2.624059	0.782891
28	1	-8.852302	-0.074154	-1.108631
29	6	-9.677133	-1.178331	0.535149
30	6	-8.323337	-1.238161	2.53825
31	1	-6.475138	-0.16232	2.461952
32	1	-6.512082	3.545973	-3.730764
33	6	-7.834814	4.210785	-2.180164
34	1	-9.073448	4.603524	-0.448117
35	1	-10.561963	-1.454767	-0.02186
36	6	-9.488489	-1.609585	1.852234
37	1	-8.194143	-1.571331	3.560715
38	6	2.459539	-0.239936	0.236912
39	6	2.864688	1.110047	0.176812
40	6	3.453675	-1.244944	0.270667
41	6	4.209697	1.428695	0.148399
42	1	2.116947	1.886187	0.135071
43	6	4.79127	-0.917754	0.243642
44	1	3.151737	-2.284148	0.329828
45	6	5.20373	0.430554	0.180504
46	1	4.502028	2.469011	0.083541
47	1	5.532131	-1.705606	0.284294
48	7	6.564634	0.764352	0.14878
49	6	7.509633	-0.107667	-0.465148
50	6	7.028127	1.980744	0.725933
51	6	7.256317	-0.659584	-1.731526
52	6	8.709607	-0.419603	0.177211

53	6	7.927021	2.79716	0.036217
54	6	6.603471	2.373479	2.005896
55	6	8.175155	-1.505419	-2.326898
56	1	6.333289	-0.419876	-2.245315
57	6	9.644141	-1.262098	-0.421131
58	1	8.915752	-0.003723	1.156039
59	1	8.261823	2.507013	-0.952311
60	6	8.398536	3.980731	0.600557
61	6	7.059255	3.552939	2.567753
62	1	5.915878	1.744031	2.557609
63	6	9.379129	-1.815109	-1.678231
64	1	7.986131	-1.933658	-3.303607
65	1	10.560658	-1.488046	0.106654
66	1	9.090509	4.591387	0.036704
67	6	7.962237	4.368692	1.871259
68	1	6.739186	3.858665	3.556354
69	8	-8.181766	5.279008	-2.948437
70	8	-10.367027	-2.379698	2.54842
71	6	-9.128582	6.210434	-2.438129
72	1	-8.765658	6.68484	-1.520285
73	1	-9.247231	6.966268	-3.212259
74	1	-10.094999	5.731997	-2.246901
75	6	-11.556181	-2.818541	1.901302
76	1	-11.327895	-3.43505	1.025706
77	1	-12.08868	-3.418891	2.636524
78	1	-12.18275	-1.971679	1.601788
79	8	8.353635	5.503489	2.512243
80	8	10.216462	-2.650915	-2.348717
81	6	9.264305	6.379589	1.858205
82	1	8.843657	6.766202	0.92398
83	1	9.428776	7.204859	2.548508
84	1	10.218278	5.882219	1.653307
85	6	11.442024	-3.031888	-1.733903
86	1	11.266382	-3.562079	-0.792109
87	1	11.931096	-3.701264	-2.439074
88	1	12.084142	-2.163298	-1.55292
89	15	-0.012822	-4.454291	-0.212164
90	9	-1.170686	-3.497744	0.553768
91	9	1.147165	-3.504871	0.559282
92	9	-0.006617	-3.342336	-1.429619
93	9	1.157514	-5.298647	-0.941388
94	9	-1.184977	-5.291386	-0.94692
95	9	-0.018908	-5.452792	1.060418

1b with the 6-31G(d) basis set

Center Number	Atomic Number	Coordinates (Å)		
		X	Y	Z
1	8	0.000013	0.533691	-0.000079
2	7	-1.151396	-1.465557	-0.014383
3	7	1.151422	-1.465555	0.014223
4	6	0.000012	-0.690226	-0.000069
5	1	-1.041351	-2.470073	-0.003104
6	1	1.041378	-2.470072	0.003008

7	6	2.494547	-1.033935	0.039814
8	6	3.48746	-2.026429	0.046136
9	6	2.888583	0.312861	0.059518
10	6	4.835761	-1.693181	0.080911
11	1	3.201361	-3.077192	0.039024
12	1	2.137004	1.089042	0.047907
13	6	4.242366	0.638368	0.072338
14	6	5.238285	-0.349075	0.088154
15	1	5.584583	-2.478467	0.098187
16	1	4.532961	1.684233	0.07376
17	7	6.616716	-0.001338	0.112243
18	6	7.068385	1.040322	0.967063
19	6	7.535206	-0.695704	-0.719504
20	6	6.569686	1.164088	2.273401
21	6	8.023287	1.969669	0.526186
22	6	8.806456	-1.056937	-0.245582
23	6	7.193339	-1.038099	-2.037545
24	1	5.838348	0.448496	2.635777
25	6	7.008756	2.193264	3.102275
26	6	8.46668	2.981902	1.3739
27	1	8.419178	1.89189	-0.481489
28	1	9.090386	-0.801198	0.770397
29	6	9.702778	-1.730871	-1.0713
30	6	8.093929	-1.728804	-2.84451
31	1	6.220198	-0.756005	-2.427026
32	1	6.607262	2.264791	4.111036
33	6	7.965937	3.122877	2.674681
34	1	9.209927	3.687217	1.007913
35	1	10.68288	-1.9948	-0.679224
36	6	9.366671	-2.089534	-2.383447
37	1	7.804236	-1.977747	-3.863414
38	6	-2.494526	-1.033943	-0.039907
39	6	-2.888571	0.312848	-0.059789
40	6	-3.487435	-2.026442	-0.046004
41	6	-4.242357	0.638346	-0.072551
42	1	-2.136996	1.089035	-0.048352
43	6	-4.83574	-1.693207	-0.080729
44	1	-3.20133	-3.077203	-0.038757
45	6	-5.238272	-0.349104	-0.088134
46	1	-4.532957	1.684209	-0.074117
47	1	-5.584558	-2.4785	-0.097837
48	7	-6.616704	-0.001369	-0.112191
49	6	-7.535169	-0.695624	0.719675
50	6	-7.068411	1.040156	-0.967158
51	6	-7.193262	-1.037868	2.037746
52	6	-8.80645	-1.056881	0.245851
53	6	-8.023251	1.969613	-0.526351
54	6	-6.569808	1.163692	-2.273543
55	6	-8.09384	-1.728453	2.844829
56	1	-6.220103	-0.755749	2.427161
57	6	-9.702755	-1.730694	1.071684
58	1	-9.090418	-0.801261	-0.770148
59	1	-8.419059	1.892006	0.48137
60	6	-8.466677	2.981715	-1.374189

61	6	-7.008913	2.192756	-3.102555
62	1	-5.838517	0.448022	-2.635859
63	6	-9.366608	-2.08921	2.38386
64	1	-7.804112	-1.977278	3.863751
65	1	-10.68288	-1.994645	0.679675
66	1	-9.209872	3.687116	-1.008257
67	6	-7.966028	3.122459	-2.675041
68	1	-6.607494	2.264112	-4.111357
69	6	-10.329291	-2.857247	3.259035
70	1	-10.215671	-3.942299	3.128194
71	1	-10.165127	-2.641479	4.320502
72	1	-11.370542	-2.611594	3.023182
73	6	-8.419556	4.249339	-3.57337
74	1	-9.443782	4.56068	-3.341019
75	1	-7.778829	5.135031	-3.462985
76	1	-8.387585	3.958275	-4.629158
77	6	10.329364	-2.857706	-3.258493
78	1	10.215557	-3.942742	-3.127686
79	1	10.165388	-2.641898	-4.319981
80	1	11.370618	-2.612214	-3.022484
81	6	8.419409	4.249868	3.572898
82	1	9.442933	4.562515	3.339233
83	1	7.777469	5.134847	3.463856
84	1	8.389284	3.95828	4.628602

1b$^+$ with the 6-311++G(d,p) basis set

Center Number	Atomic Number	Coordinates (Å)		
		X	Y	Z
1	8	-0.000026	0.505659	-0.001845
2	7	-1.151891	-1.487517	0.004804
3	7	1.152080	-1.487394	-0.008919
4	6	0.000051	-0.709032	-0.001980
5	1	-1.040438	-2.490126	0.042871
6	1	1.040729	-2.490007	-0.047185
7	6	2.483172	-1.054610	0.012355
8	6	3.482874	-2.045353	-0.018736
9	6	2.871391	0.295814	0.066728
10	6	4.821544	-1.710598	0.005634
11	1	3.202307	-3.093939	-0.045529
12	1	2.120126	1.069392	0.072380
13	6	4.214708	0.627287	0.091863
14	6	5.218659	-0.359279	0.063680
15	1	5.569857	-2.492196	-0.001319
16	1	4.496900	1.671823	0.118074
17	7	6.575649	-0.009758	0.096904
18	6	7.009304	1.135404	0.823544
19	6	7.541510	-0.795354	-0.594094
20	6	6.522778	1.387647	2.113016
21	6	7.945011	2.015360	0.262610
22	6	8.753214	-1.130008	0.024813
23	6	7.302988	-1.225357	-1.906288
24	1	5.818483	0.700953	2.566814
25	6	6.958842	2.503942	2.815781

26	6	8.373954	3.123789	0.980009
27	1	8.327388	1.829490	-0.733631
28	1	8.947404	-0.801057	1.038366
29	6	9.698311	-1.883620	-0.658848
30	6	8.257208	-1.981753	-2.574925
31	1	6.381255	-0.948170	-2.403562
32	1	6.581338	2.676072	3.818029
33	6	7.888941	3.395785	2.266814
34	1	9.094717	3.796864	0.527903
35	1	10.629305	-2.136773	-0.163020
36	6	9.471199	-2.329377	-1.967859
37	1	8.062789	-2.292838	-3.595852
38	6	-2.483028	-1.054830	-0.015703
39	6	-2.871365	0.295557	-0.070106
40	6	-3.482643	-2.045629	0.016340
41	6	-4.214722	0.626950	-0.094243
42	1	-2.120149	1.069180	-0.076455
43	6	-4.821349	-1.710956	-0.007042
44	1	-3.201989	-3.094191	0.043189
45	6	-5.218592	-0.359668	-0.065011
46	1	-4.496992	1.671465	-0.120436
47	1	-5.569617	-2.492591	0.000698
48	7	-6.575642	-0.010276	-0.097063
49	6	-7.540753	-0.795770	0.595111
50	6	-7.010101	1.134764	-0.823415
51	6	-7.300956	-1.225261	1.907229
52	6	-8.752975	-1.130831	-0.022573
53	6	-7.945334	2.014710	-0.261658
54	6	-6.524872	1.386897	-2.113392
55	6	-8.254455	-1.981560	2.577020
56	1	-6.378801	-0.947771	2.403548
57	6	-9.697315	-1.884354	0.662215
58	1	-8.948129	-0.802260	-1.036066
59	1	-8.326721	1.828917	0.734975
60	6	-8.375064	3.123023	-0.978750
61	6	-6.961713	2.503092	-2.815851
62	1	-5.820983	0.700200	-2.567815
63	6	-9.468927	-2.329600	1.971189
64	1	-8.059054	-2.292255	3.597876
65	1	-10.628717	-2.137850	0.167328
66	1	-9.095431	3.796096	-0.526007
67	6	-7.891336	3.394921	-2.266068
68	1	-6.585212	2.675139	-3.818491
69	6	-10.487054	-3.172329	2.694926
70	1	-10.326798	-4.236522	2.489504
71	1	-10.422719	-3.035500	3.776288
72	1	-11.503774	-2.929562	2.379253
73	6	-8.342760	4.617726	-3.021765
74	1	-9.397281	4.834250	-2.836705
75	1	-7.770582	5.499016	-2.711641
76	1	-8.202016	4.497363	-4.097522
77	6	10.490092	-3.172196	-2.690409
78	1	10.328600	-4.236464	-2.486344
79	1	10.427958	-3.034289	-3.771769

80	1	11.506376	-2.930511	-2.372534
81	6	8.339473	4.618710	3.022849
82	1	9.393719	4.836310	2.837526
83	1	7.766333	5.499578	2.713295
84	1	8.199194	4.497777	4.098607

1b^{+}-PF_6^{-} with the 6-311++G(d,p) basis set

Center Number	Atomic Number	Coordinates (Å)		
		X	Y	Z
1	8	0.003084	1.479982	-0.124256
2	7	-1.137689	-0.520887	-0.275158
3	7	1.150263	-0.519282	-0.247241
4	6	0.005093	0.261997	-0.206241
5	1	-1.02405	-1.533396	-0.33483
6	1	1.039747	-1.532245	-0.306661
7	6	2.465836	-0.0939	-0.170346
8	6	3.462513	-1.097711	-0.175869
9	6	2.865725	1.257024	-0.088514
10	6	4.797399	-0.768204	-0.102724
11	1	3.16302	-2.138395	-0.218932
12	1	2.116733	2.032987	-0.096504
13	6	4.20808	1.577547	-0.015687
14	6	5.204428	0.580886	-0.020096
15	1	5.539734	-1.555514	-0.089262
16	1	4.498416	2.619329	0.03077
17	7	6.562695	0.91811	0.056694
18	6	6.984643	2.065735	0.787987
19	6	7.545682	0.1152	-0.591689
20	6	6.474027	2.32354	2.066301
21	6	7.931403	2.942998	0.246273
22	6	8.724762	-0.238075	0.074494
23	6	7.353561	-0.320288	-1.908745
24	1	5.755072	1.641427	2.503781
25	6	6.896255	3.442324	2.774759
26	6	8.350717	4.052944	0.970835
27	1	8.33599	2.751783	-0.740285
28	1	8.884994	0.093879	1.093145
29	6	9.683998	-1.013448	-0.566998
30	6	8.31729	-1.102858	-2.533457
31	1	6.452449	-0.039173	-2.440304
32	1	6.494427	3.618997	3.767167
33	6	7.839888	4.33024	2.244334
34	1	9.084294	4.721552	0.532152
35	1	10.589508	-1.279862	-0.031704
36	6	9.49965	-1.467595	-1.878175
37	1	8.151131	-1.425813	-3.555987
38	6	-2.455303	-0.097374	-0.240049
39	6	-2.859737	1.251791	-0.150302
40	6	-3.449876	-1.101708	-0.298012
41	6	-4.204157	1.569856	-0.115954
42	1	-2.111678	2.026391	-0.090177
43	6	-4.786767	-0.774689	-0.263995
44	1	-3.147827	-2.139201	-0.38132

45	6	-5.198534	0.57243	-0.170173
46	1	-4.496754	2.608257	-0.02759
47	1	-5.528211	-1.560675	-0.323136
48	7	-6.55896	0.90585	-0.129204
49	6	-7.502056	0.024492	0.476054
50	6	-7.023771	2.131056	-0.688198
51	6	-7.233564	-0.560721	1.719652
52	6	-8.717004	-0.257412	-0.158784
53	6	-7.93621	2.928697	0.012419
54	6	-6.591067	2.546958	-1.953408
55	6	-8.158671	-1.418888	2.301952
56	1	-6.303487	-0.337489	2.22827
57	6	-9.6367	-1.110444	0.440769
58	1	-8.936074	0.190777	-1.120399
59	1	-8.28072	2.615275	0.990472
60	6	-8.397991	4.115866	-0.544202
61	6	-7.054866	3.741092	-2.492986
62	1	-5.899804	1.928616	-2.513212
63	6	-9.376344	-1.713723	1.676795
64	1	-7.932945	-1.858631	3.267892
65	1	-10.571353	-1.319397	-0.069372
66	1	-9.103585	4.720546	0.016258
67	6	-7.964572	4.550649	-1.802378
68	1	-6.713376	4.040942	-3.478399
69	6	-10.360377	-2.670366	2.302179
70	1	-10.133744	-3.704159	2.018954
71	1	-10.328942	-2.618093	3.392945
72	1	-11.382451	-2.459261	1.980292
73	6	-8.442077	5.858164	-2.383369
74	1	-9.449483	6.103553	-2.039787
75	1	-7.78476	6.682544	-2.085202
76	1	-8.452887	5.828807	-3.475277
77	6	10.525749	-2.342581	-2.553611
78	1	10.294602	-3.402937	-2.403418
79	1	10.553442	-2.165398	-3.631284
80	1	11.526618	-2.166121	-2.153689
81	6	8.272309	5.556218	3.009269
82	1	9.289518	5.853642	2.744984
83	1	7.615556	6.405774	2.791323
84	1	8.237086	5.385987	4.087758
85	15	-0.00817	-4.303937	0.178275
86	9	1.173519	-3.354396	-0.560933
87	9	-1.143088	-3.351849	-0.627937
88	9	-0.042412	-3.183973	1.389268
89	9	-1.20143	-5.138178	0.880469
90	9	1.140181	-5.140683	0.94841
91	9	0.027379	-5.307472	-1.088812

Table S2: Selected vertical excitation energies for MV species predicted by TD-DFT.

1a$^+$-PF_6^- with the 6-311++G(d,p) basis set in the gas phase

#	*f*	eV	nm	transition[a]	coefficients
1	0.6739	0.6777	1829.55	210β→211β	1.06221
2	0.0138	1.5565	796.57	209β→211β	0.99133
3	0.0774	1.8280	678.24	208β→211β	0.99207
4	0.0459	2.1119	587.07	207β→211β	0.75199
5	0.0574	2.1123	586.96	206β→211β	0.75172

[a] Main contributions (> 0.75) are shown. Total number of states in the calculation: 30.

1b$^+$-PF_6^- with the 6-311++G(d,p) basis set in gas phase

#	*f*	eV	nm	transition[a]	coefficients
1	0.6589	0.7104	1745.31	194β→195β	1.05490
2	0.0140	1.6550	749.14	193β→195β	0.98936
3	0.0835	1.9245	644.25	192β→195β	0.98950
4	0.0029	2.2265	556.85	191β→195β	0.80406
5	0.0054	2.2297	556.05	190β→195β	0.80098

[a] Main contributions (> 0.80) are shown. Total number of states in the calculation: 30.

3. Electrochemical investigation

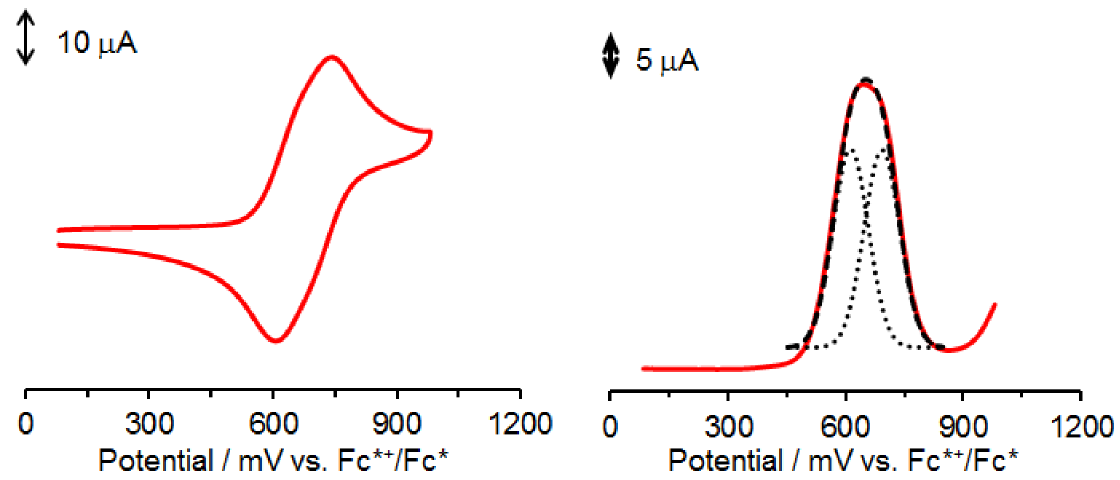

Figure S9: Cyclic voltammograms (left) and differential pulse voltammograms (right) of **1a** (1.0 mM) in MeCN/CH_2Cl_2 9:1 containing *n*-Bu_4NPF_6 (0.10 M). Scan rate: 100 mV s^{-1}. For the differential pulse voltammogram, Gaussian deconvolution (black dotted line) and the sum (black dashed line) are also shown.

Table S3: Electrochemical data for **1a** and **MeO-TPD**.[a]

Compounds	Solvent	$E_{1/2}^{1}$	$E_{1/2}^{2}$	$\Delta E_{1/2}^{b}$	K_c^{c}
**1a**	$MeCN/CH_2Cl_2$ (9/1)	631	712	81	23
**MeO-TPD**	$MeCN/CH_2Cl_2$ (9/1)	679	837	158	469
	CH_2Cl_2	663	890	227	1.94×10^4

[a]In the presence of 0.1M n-Bu_4NPF_6. Potentials in mV vs. Fc^{*+}/Fc^* (Fc^* = decamethylferrocene). [b]$\Delta E_{1/2}$ = potential difference between two redox processes. [c]Comproportionation constants obtained from $K_c = \exp(\Delta E_{1/2} F/RT)$.

Table S4: Electrochemical data for **1** and **MeO-TPD**.[a]

Compounds	Solvent	X^-	$E_{1/2}^{1}$	$E_{1/2}^{2}$	$\Delta E_{1/2}$
**1a**	CH_2Cl_2	PF_6^-	46	140	94
	CH_2Cl_2	$BArF_4^-$	56	122	66
	$MeCN/CH_2Cl_2$ (9/1)	PF_6^-	119	200	81
**1b**	CH_2Cl_2	PF_6^-	136	280	144
	CH_2Cl_2	$BArF_4^-$	166	273	107
**Ph1b**	CH_2Cl_2	PF_6^-	164	-	-
**MeO-TPD**	CH_2Cl_2	PF_6^-	103	330	227
	$MeCN/CH_2Cl_2$ (9/1)	PF_6^-	167	325	158

[a]In the presence of 0.1 M n-Bu_4NX. Potentials in mV vs. Fc^+/Fc. In CH_2Cl_2, the Fc^+/Fc couples are 560 and 618 mV *vs* Fc^{*+}/Fc^*, and 664 and 568 mV *vs* Ag/AgCl in CH_2Cl_2 containing n-Bu_4NPF_6 and n-Bu_4NBArF_4, respectively. In $MeCN/CH_2Cl_2$ 9:1, the Fc^+/Fc couples are 512 mV *vs* Fc^{*+}/Fc^* and 435 mV *vs* Ag/AgCl.

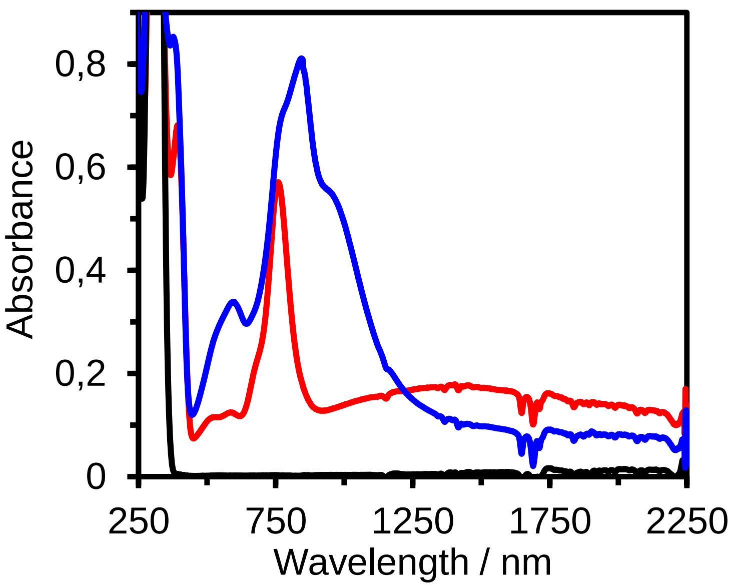

Figure S10: UV–vis-NIR spectra of a CH_2Cl_2 solution containing **1b** (4.5×10^{-4} M) and *n*-Bu_4NPF_6 (0.10 M) before and after the controlled potential electrolysis (at 20 mV (black), 755 mV (red), and 1029 mV (blue) vs. Fc^{*+}/Fc^*, respectively).

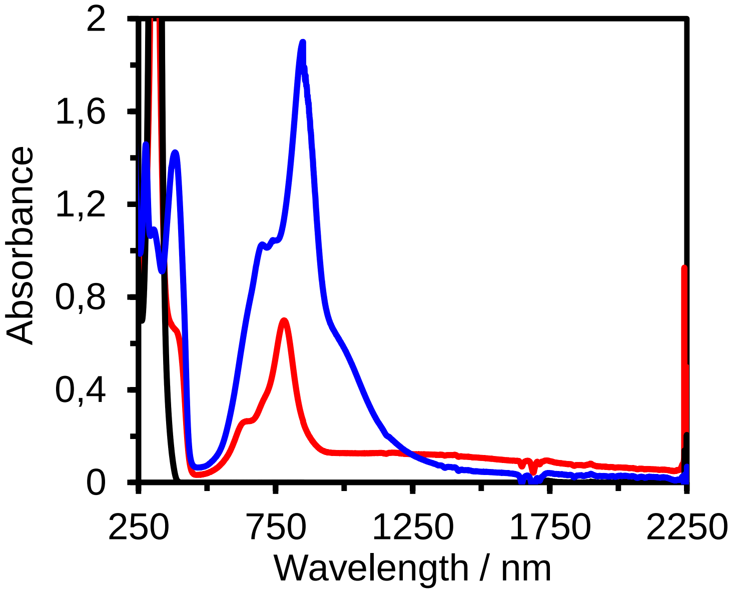

Figure S11: UV–vis-NIR spectra of a CH_2Cl_2 solution containing **1a** (4.3×10^{-4} M) and *n*-Bu_4NPF_6 (0.10 M) before and after the controlled potential electrolysis (at 33 mV (black), 642 mV (red), and 779 mV (blue) vs. Fc^{*+}/Fc^*, respectively).

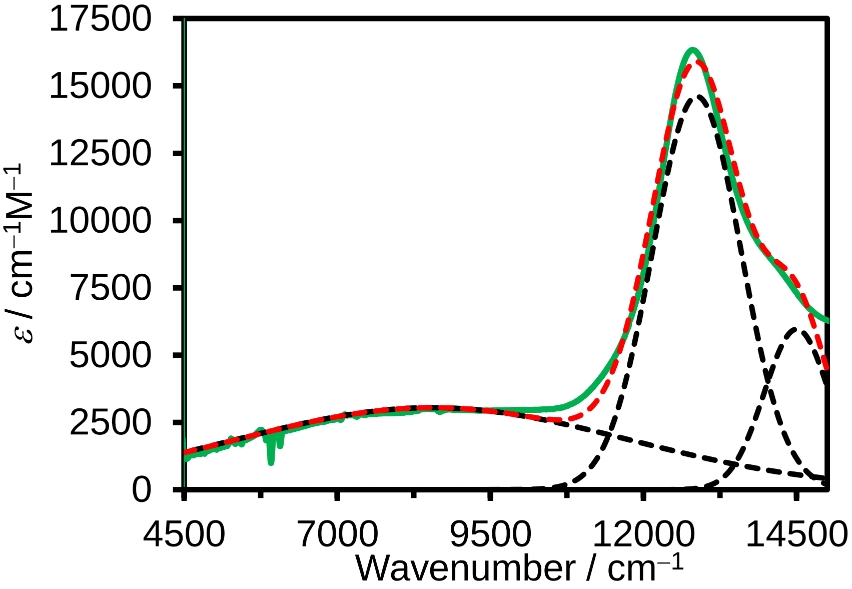

Figure S12: Vis-NIR spectra of **1a**$^{+}$ (green line) prepared by bulk electrolysis, with Gaussian deconvolutions (black broken lines) and the sum (red broken line).
